# Evaluation and Selection of HazMat Transportation Alternatives: A PHFLTS- and TOPSIS-Integrated Multi-Perspective Approach

**DOI:** 10.3390/ijerph16214116

**Published:** 2019-10-25

**Authors:** Zhen-Song Chen, Min Li, Wen-Tao Kong, Kwai-Sang Chin

**Affiliations:** 1 School of Civil Engineering, Wuhan University, Wuhan 430072, China; zschen@whu.edu.cn (Z.-S.C.); lm6224@whu.edu.cn (M.L.); 2 Department of Systems Engineering and Engineering Management, City University of Hong Kong, Hong Kong; mekschin@cityu.edu.hk

**Keywords:** hazardous materials transportation, alternative evaluation, proportional hesitant fuzzy linguistic term set, entropy measure, multi-criteria group decision-making

## Abstract

Outsourcing the hazardous materials (HazMat) transportation is an effective way for manufacturing enterprises to avoid risks and accidents as well as to retain sustainable development in economic growth and social inclusion while not bringing negative impacts on the public and the environment. It is imperative to develop viable and effective approaches to selecting the most appropriate HazMat transportation alternatives. This paper aims at proposing an integrated multi-criteria group decision making approach that combines *proportional hesitant fuzzy linguistic term set* (PHFLTS) and the *Technique for Order of Preference by Similarity to Ideal Solution* (TOPSIS) to address the problem of HazMat transportation alternative evaluation and selection. PHFLTSs are adopted to represent the congregated individual evaluations in a bid to avoid information loss and increase the reliability of results. Two weight assignment models are then proposed to determine the comprehensive weights of experts and criteria. Furthermore, several novel manipulations of PHFLTS are also defined to enrich its applicability. The TOPSIS method is subsequently extended to the context of PHFLTSs to rank alternatives and choose the best one. Eventually, the feasibility and validity of the proposed approach are verified by a practical case study of a HazMat transportation alternative evaluation and selection decision and further comparison analyses.

## 1. Introduction

With the development of industrialization and socialization, the production and transportation requirements of hazardous materials (HazMat) are increasing synchronously. The usage of HazMat could bring the growth of economy and the facilitation of daily life, while also leads to catastrophic losses in life, economy, environment, and society once accidents happened during the process of HazMat transportation since HazMat are generally related to five harmful properties, which exactly mean toxicity, ignitability, corrosivity, reactivity and infectivity [1]. Specifically, the HazMat transportation accidents may harm the human life and cause social panic because the harmful characteristics of hazardous substances; the economic losses occurred because the traffic congestion, materials loss and production delays resulted by HazMat transportation accidents; and the air pollution, water pollution, and land pollution may be caused by the toxic releases in the accidents. All of the previously discussed negative consequences can generate huge social and environmental cost, which will impede the sustainable development of the society that are instrumental to economic growth and social inclusion [2,3,4]. Therefore, sustainability of HazMat transportation is a critical problem in satisfying the development requirements of society and economy without bringing negative influences on the natural and social environment. Different from the transportation of regular goods, HazMat transportation requires special-purpose equipment and qualified operators. It is therefore difficult, costly, and risky for many HazMat manufacturing enterprises to transport the HazMat by themselves. To allocate the limited resources to competitive business for profit growth, risk aversion and long-term sustainable development, many HazMat manufacturing enterprises choose to outsource their HazMat transportation business to professional HazMat transportation company. Safety guarantee or risk decreasing and pollution prevention including air, water, and land are two main indicators for sustainable rating [5]. Therefore, evaluation and selection of HazMat transportation alternatives based on risk evaluation have become a highlighted problem both for HazMat manufacturing enterprises and management authorities of government during the decision-making process of transportation outsource. To support the decision, it is important to develop a scientific method for evaluating the HazMat transportation risk and selecting an appropriate transportation alternative. Further, since many risk factors are involved in the process of HazMat transportation, risk evaluation and alternative selection of HazMat transportation could be regarded as a multiple criteria decision-making (MCDM) problem, and, further, a multiple criteria group decision-making (MCGDM) given that several experts are invited to initiate multiple perspectives [5].

Risk management of HazMat transportation has attracted numerous concerns to the researchers and practitioners, and various approaches have been proposed for the past decades. Erkut and Verter [6] developed a quantitative comprehensive risk framework for HazMat road transportation based on an extended risk assessment approach by considering the population density. However, the accuracy of the results of the proposed approach is dependent largely on the data used. Bonvicini et al. [7] put forward a fuzzy logic-based method for evaluating the risk of HazMat transportation, in which fuzzy numbers were used to manage the uncertainties involved in the risk estimates. Fabiano et al. [8] proposed a risk evaluation and decision-making method to analyze the influence of risk factors such as weather and road condition on HazMat transportation risk based on the accidents statistical data. Based on the historical incident data, Clark and Besterfield-Sacre [9] came up with a risk evaluation model on the basis of Bayesian network for assessing the risk. However, a conspicuous disadvantage of this model is that the quality of the proposed model relied largely on the accuracy of the input data. Similarly, Qiao et al. [10] also developed an empirical data-based risk estimation model for HazMat transportation. Liu et al. [11] regarded the risk management of HazMat transportation as an MCDM problem and developed an optimal investment model that incorporated the risk decrease strategies. Bodar et al. [12] studied the risk management problem of hazardous substances in circular economy and presented a new view on integrating sustainability with safety based on the analysis of several practical cases. Yoo and Choi [13] proposed a geographic information system (GIS)-based risk analysis method to assist experts to establish the emergency response and risk communication plan when hazardous chemicals leakage accidents happened. Wey [14] proffered an integrated approach based on fuzzy Delphi method (FDM) and dynamic network process (DNP) for identifying sustainable urban transportation planning strategies for improving quality of life. One of the effective measures mentioned in this study is to decrease the risk of transportation. Two limitations of HazMat transportation could be summarized based on the previous analysis. On the one hand, it can be seen that most of the existing methods for risk evaluation of HazMat transportation are largely dependent on input empirical data. However, collecting the required data is extremely difficult for many developing countries such as China as there is no special database to record the related accidents data. For overcoming the data-dependence, an effective way is to fully make use of the experiences and knowledge of experts in the field. Therefore, this paper is poised to propose a novel method for risk evaluation and alternative selection of HazMat transportation from a new perspective of MCGDM problem. On the other hand, although it is well-known that sustainable development is one of the most important goals in society practice and research [15] and the importance of sustainable transportation of HazMat has been identified, no specific study aims at providing the HazMat manufacturing a scientific decision support approach for selecting transportation alternatives from the perspective of sustainability. Therefore, this study intends to bridge the research gap by developing a systematic decision-making framework for HazMat manufacturing company. This presents another motivation of this research.

Alternative evaluation and selection is often conceptualized as an MCDM/MCGDM problem, in which the alternatives are evaluated by a group of experts with respect to a number of criteria. Based on the evaluation information given by experts and a certain MCDM method, a ranking of alternatives and the best one could be determined. There are many methods for solving the MCDM/MCGDM problem. For example, Garg and Kumar [16] extended the traditional TOPSIS method into interval-valued intuitionistic fuzzy (IVIF) set environment, and then used the proposed method to help the government selecting the best company for a project. Garg and Kaur [17] proposed an MCGDM method for solving the problem of selecting the best candidate for a new project by integrating the TOPSIS with cubic intuitionistic fuzzy sets. Moreover, numerous methods have been developed to solve the alternatives evaluation and selection problem in various fields. Ju et al. [18] combined analytic network process (ANP) and decision-making trial and evaluation laboratory (DEMATEL) with technique for order preference by similarity to an ideal solution (TOPSIS) under 2-tuple linguistic environment to manage the emergency alternative evaluation and selection problem. Mohagheghi et al. [19] proposed an interval-valued fuzzy sets (IVFSs)-based approach to address the evaluation and selection of sustainable transport projects. Bandeira et al. [20] brought forward a fuzzy MCDM model to select alternative configurations for sustainable urban freight transportation. Büyüközkan et al. [21] presented a group decision-making technique based on integrated intuitionistic fuzzy Choquet integral for selecting sustainable urban transportation alternatives. Chen and Yu [22] propounded an approach incorporating the extension entropy, best-worst method (BWM) and intuitionistic fuzzy weighted averaging operator to select emergency alternative. Although the existing methods could provide certain references, they cannot perfectly fit the application of evaluation and selection of HazMat transportation alternatives from two main aspects. On the one hand, when choosing the tool to represent evaluation information of experts, three basic situations should be taken into account. Firstly, considering the vagueness and uncertainty characteristics of HazMat transportation risk as well as the habits of human thinking, it is more appropriate to use linguistic terms or expressions to represent the evaluation information given by experts. Secondly, because of the limitation of time, knowledge and experiences, experts may hesitate among several linguistic terms. Therefore, hesitant fuzzy linguistic term set (HFLTS) should be adopted to express the hesitancy of experts. Thirdly, as previously stated, since a group of experts would be included in the decision-making, it is important to avoid information loss when aggregating the individual evaluation of each expert [23]. Based on the analysis, proportional HFLTS (PHFLTS) was introduced into the study for information representation. On the other hand, evaluation and selection of alternatives is an MCGDM problem, in which both the weights of experts and criteria would influence the decision results [24,25]. Therefore, it is necessary to derive the weights of experts and criteria comprehensively. This is another motivation of our research. To this end, this paper constructs two weighting determination models to derive the weights of experts and criteria. Specifically, a distance measure for PHFLTSs was defined first, and then an expert weight determination model was proposed on the basis of the distance measure concerning each expert with others. Meanwhile, another weighting model used for determining the weights of criteria was proposed as per a newly defined entropy measure for PHFLTSs. In summary, we discuss in this study the problem of alternative evaluation and selection of HazMat transportation via proposing a novel MCGDM approach under proportional hesitant fuzzy linguistic (PHFL) environment considering the characteristics of HazMat transportation risk and the habits of human thinking. Compared with the previous related research, the advantages and novelty of our proposed method can be summarized into two main aspects. On the one hand, we use HFLTSs and PHFLTSs to represent and aggregate the evaluations information, which can retain the information as much as possible as further increase the reliability of the results. On the other hand, the comprehensive weights of criteria and experts are taken into account simultaneously, which can also increase the accuracy of the obtained results.

The main difficulties and challenges of the research can be summarized as follows: (a) How can as much of the original information given by experts as possible be retained when the information processing techniques are faced with certain fuzziness and randomness? (b) How can the weights of experts and criteria be determined reasonably in the context of MCGDM? (c) How can an appropriate ranking method be chosen for the determination of the best hazmat transportation alternative? To answer these questions, this paper exerts the following efforts. Firstly, we adopt HFLTS to express the evaluation of experts and then the PHFLTS to integrate the evaluation information. It facilitates not only the expressions of experts but also retains to the utmost degree the original information without manipulations introducing information loss or distortion. Secondly, to determine the weights of experts and criteria, two weight assignment models based on the entropy and similarity measures for PHFLTS are proposed. In contrast to other weights determination methods such as subjective weighting methods AHP, scoring points and Delphi methods, or objective weighting methods (e.g., deviation and CRITIC methods), the proposed comprehensive weight assignment models use adequately the subjective and objective weights and, therefore, are more reliable. Eventually, in terms of the selection of the most appropriate HazMat transportation alternative, an integrated proportional hesitant fuzzy linguistic TOPSIS (PHFL-TOPSIS) method is proposed on the basis of the distance measure of PHFLTS. This integrated approach takes full advantage of the PHFLTS (e.g., eliminating information loss and distortion and increasing the reliability of the decision outcomes) and the TOPSIS method [26]. Therefore, the final results obtained by the proposed PHFLTS- and TOPSIS-integrated multi-perspective approach are deemed to be reliable and accuracy.

The remainder of this paper is organized as follows. Section 2 reviews some basic concepts and operations related to this study. Some novel operations and information measures for PHFLTSs are proposed in Section 3. Two weighting models based on the defined distance measure and entropy measure for PHFLTSs are also established in this section to determine the objective weights of experts and criteria respectively. Section 4 presents the proposed MCGDM approach that combined TOPSIS with PHFLTSs for alternative evaluation and selection in detail. Section 5 provides a practical case study of an alternative evaluation and selection of HazMat transportation accompanied by comparison analysis to demonstrate the superiority of the proposed integrated MCGDM method. Section 6 summarizes the study and points out the future research directions.

## 2. Preliminaries

This section recalls some basic concepts, definitions, operations and properties of HFLTSs and PHFLTSs.

### 2.1. Fuzzy Linguistic Approach

To deal with the situation where the evaluation information cannot be represented by numbers but can be appropriately described in a qualitative manner, the fuzzy linguistic approach that uses fuzzy set theory is proposed, in which the uncertain information was expressed as linguistic variables [27,28]. A linguistic variable means that“*the values of the variable are not numbers but words or sentences in a natural or artificial language*”. The definition of a linguistic variable is as follows.

**Definition** **1** ([29])**.** *A linguistic variable is characterized by a quintuple $\left(H,T,U,G,M\right)$, in which H represents the name of the variable; T represents the term set of H; U denotes the universe where the value of each fuzzy variable comes from; G is a syntactic rule for generating the names of values of H; and M is a semantic rule for the association of its meaning with each H.*

It is important to select the appropriate linguistic descriptors for the linguistic term set (LTS) and their semantics in order to deal with linguistic variables. Two approaches as shown below are usually used to select the linguistic descriptors.

(1) *An ordered structure approach*: In this approach, the LTS is defined based on an ordered structure that provides the term set that is distributed on a total ordered scale. Generally, the number of elements, also known as cardinality, of a LTS is an odd number, the central linguistic term represents a meaning of “indifference”, and all other linguistic terms are distributed symmetrically around the central linguistic term. Let $S=\left\{s_{0},s_{1},\cdots ,s_{g}\right\}$ be a LTS whose granularity g+1 is an odd number. Then, the following properties need to be satisfied:
  (a) (Orderliness) $s_{i}\leq s_{j}$, if i≤j;
  (b) (Maximization operator) $\max\left(s_{i},s_{j}\right)=s_{i}$, if $s_{i}\geq s_{j}$;
  (c) (Minimization operator) $\min\left(s_{i},s_{j}\right)=s_{i}$, if $s_{i}\leq s_{j}$; and
  (d) (Negation operator) $Neg\left(s_{i}\right)=s_{j}$, where j=g−i.
(2) *A context-free grammar approach*: In this approach, the LTS is defined based on a context-free grammar, which uses words or sentences in a natural or artificial language to express the linguistic terms. The context-free grammar could be represented by a quaternary $\left(V_{N},V_{T},I,P\right)$, where $V_{N}$ represents the set of nonterminal symbols, $V_{T}$ represents the set of terminals’ symbols, *I* represents the starting symbol, and *P* represents the production rules. Further, for dealing with hesitant situations in group decision-making (GDM), Rodríguez et al. [30] proposed an extended context-free grammar $G_{H}$ to generate comparative linguistic expressions. The definition is as follows.

**Definition** **2** ([30])**.** *Let $G_{H}=\left(V_{N},V_{T},I,P\right)$ be a context-free grammar and $S=\left\{s_{0},s_{1},\cdots ,s_{g}\right\}$ be an LTS. Then,*

$\begin{array}{c} V_{N}=\left\{\langle primary\;\;term\rangle ,\langle composite\;\;term\rangle ,\langle unary\;\;relation\rangle ,\langle binary\;\;relation\rangle ,\langle conjunction\rangle \right\}\\ V_{T}=\left\{lower\;\;than,greater\;\;than,at\;\;least,at\;\;most,between\;\;and,s_{0},s_{1},\cdots ,s_{g}\right\}\\ I\in V_{N} \end{array}$

The production rules are defined in an extended Backus–Naur form such that the brackets enclose optional elements and the symbol ∣ indicates alternative elements [31]. For the context-free grammar $G_{H}$, the production rules are as follows:

$\begin{array}{c} P=\left\{I::==\left.\langle primary\;\;term\rangle \right|\langle composite\;\;term\rangle \right.\\ \langle composite\;\;term\rangle ::==\langle unary\;\;relation\rangle \left.\langle primary\;\;term\rangle \right|\langle binary\;\;relation\rangle \\ \langle primary\;\;term\rangle \langle conjunction\rangle \langle primary\;\;term\rangle \\ \langle primary\;\;term\rangle ::==\left.s_{0}\right|\left.s_{1}\right|\left.\cdots \right|s_{g}\\ \langle unary\;\;relation\rangle ::==\left.lower\;\;than\right|\left.greater\;\;than\right|\left.at\;\;least\right|at\;\;most\\ \langle binary\;\;relation\rangle ::==between\\ \langle conjunction\rangle ::==and \end{array}$

In GDM settings, the experts may hesitate among serval alternatives for various reasons. Therefore, the hesitancy of experts should be taken into account. Hesitant fuzzy set (HFS) and its various extensions [32] are good at dealing with this situation. Motivated by the idea of fuzzy linguistic approach and HFS, Rodríguez et al. [30] proposed the concept of HFLTS for dealing with the situation where experts are hesitant among several linguistic terms when evaluating a linguistic variable in a qualitative problem.

### 2.2. Hesitant Fuzzy Linguistic Term Sets

**Definition** **3** ([30])**.** *Let $S=\left\{s_{0},s_{1},\cdots ,s_{g}\right\}$ be an LTS. An HFLTS, denoted as $H_{S}$, on S is an ordered finite subset of S with consecutive linguistic terms in it.*

Sophisticated linguistic constructions capable of modeling more precisely and flexibly individual semantics are necessitated as experts judge decision problems with their own knowledge and attitudes [33,34]. Elaborating linguistic constructions has led to the introduction of context-free grammar by Rodríguez et al. [30] and further extended by Rodríguez et al. [35] in order to enrich the expression domain. The production rules in Definition 2 are built in context-free grammars, and, thus, comparative linguistic expressions can be generated, which in combination with all singletons given a predetermined LTS are routinely considered as a whole for complex linguistic constructions, and they collectively are known as generalized comparative linguistic expressions (GCLEs) [36,37]. GCLEs themselves are not directly machine manipulatable, and the approximate equivalent linguistic transformation (AppELT) developed by Rodríguez et al. [30] achieves this goal by transforming GCLEs into HFLTSs.

**Definition** **4** ([30])**.** *Let $E_{G_{H}}$ be a function that transforms the linguistic expression $ll\in S_{ll}$ obtained by $G_{H}$ into an HFLTS. S is the LTS used by $G_{H}$, and $S_{ll}$ is the expression domain generated by $G_{H}$:*

*$E_{G_{H}}:S_{ll}\to H_{S}$.*

The linguistic expressions generated by $G_{H}$ using the production rules given in Definition 2 can be transformed into an HFLTS through the following transformations:

(i) $E_{G_{H}}\left(s_{i}\right)=\left\{s_{i}\right\}$ for arbitrary $s_{i}\in S$;
(ii) $E_{G_{H}}\left(at\;\;least\;\;s_{i}\right)=\left\{\left.s_{j}\right|s_{j}\geq s_{i}\;\;and\;\;s_{j}\in S\right\}$;
(iii) $E_{G_{H}}\left(at\;\;most\;\;s_{i}\right)=\left\{\left.s_{j}\right|s_{j}\leq s_{i}\;\;and\;\;s_{j}\in S\right\}$;
(iv) $E_{G_{H}}\left(lower\;\;than\;\;s_{i}\right)=\left\{\left.s_{j}\right|s_{j}<s_{i}\;\;and\;\;s_{j}\in S\right\}$;
(v) $E_{G_{H}}\left(greater\;\;than\;\;s_{i}\right)=\left\{\left.s_{j}\right|s_{j}>s_{i}\;\;and\;\;s_{j}\in S\right\}$;
(vi) $E_{G_{H}}\left(between\;\;s_{i}\;\;and\;\;s_{j}\right)=\left\{\left.s_{k}\right|s_{i}\leq s_{k}\leq s_{j}\;\;and\;\;s_{k}\in S\right\}$.

**Definition** **5** ([30])**.** *The envelope of the HFLTS, denoted as $env\left(H_{S}\right)$, is a linguistic interval whose limits are obtained by means of upper bound (max) and lower bound (min). Hence,*

(1) $$env\left(H_{X}\right)=\left[H_{S}^{-},H_{S}^{+}\right],$$

*where the lower bound $H_{S}^{-}=\min\left\{\left.s_{i}\right|s_{i}\in H_{S}\right\}=s_{j}$ and $s_{i}\geq s_{j}$, and the upper bound $H_{S}^{+}=\max\left\{\left.s_{i}\right|s_{i}\in H_{S}\right\}=s_{j}$ and $s_{i}\leq s_{j}$.*

**Definition** **6** ([30])**.** *Based on the concept of the envelope of HFLTS, the comparison laws between two HFLTSs, $H_{S}^{1}\left(\vartheta \right)$ and $H_{S}^{2}\left(\vartheta \right)$, is defined as follows:*

*(1)*  $H_{S}^{1}\left(\vartheta \right)>H_{S}^{2}\left(\vartheta \right)\;\;iff\;\;env\left(H_{S}^{1}\left(\vartheta \right)\right)>env\left(H_{S}^{2}\left(\vartheta \right)\right)$; and
*(2)*  $H_{S}^{1}\left(\vartheta \right)=H_{S}^{2}\left(\vartheta \right)\;\;iff\;\;env\left(H_{S}^{1}\left(\vartheta \right)\right)=env\left(H_{S}^{2}\left(\vartheta \right)\right)$.

The comparison laws between two HFLTSs defined above are further accomplished following the rules of comparing any two interval values as given in the Appendix of [30]. The comparison of HFLTS envelope is de facto a stepwise rule that contain a partial-order relation and the construction of reciprocal preference degree to guarantee the reasonable comparison among a set of given HFLTSs. The first phase is to employ the second-order relation based on the center and width of the interval, and it introduces an acceptability function that indicates the grade of acceptability regarding the first interval is inferior to the second interval. The second phase further enables us to discover the reciprocal preference degree between both intervals. The method proposed by Wang et al. [38] is applied to obtain a preference relation from a vector of intervals, and it is used in BWM presented in Section 4. The detailed process can be accessed conveniently in Rodríguez et al. [30] and, therefore, are not reiterated herein.

**Definition** **7** ([39])**.** *Let $H_{S}^{1}$ and $H_{S}^{2}$ be two HFLTSs defined on the LTS S. A normalized Hamming distance measure for HFLTSs is defined as*

(2) $$d\left(H_{S}^{1},H_{S}^{2}\right)=\frac{1}{3g}\left(\left|Ind\left(H_{S}^{1-}\right)-Ind\left(H_{S}^{2-}\right)\right|+\left|Ind\left(H_{S}^{1+}\right)-Ind\left(H_{S}^{2+}\right)\right|+\left|\theta \left(H_{S}^{1}\right)-\theta \left(H_{S}^{2}\right)\right|\right),$$

*where $\theta \left(H_{S}\right)=\frac{1}{2}\left(Ind\left(H_{S}^{-}\right)+Ind\left(H_{S}^{+}\right)\right)$ represents the averaging value of $H_{S}$ and Ind( ) denotes the set of indexes of the linguistic terms in an HFLTS.*

### 2.3. Proportional Hesitant Fuzzy Linguistic Term Set

The process of integrating all the individual HFLTSs into group evaluations is expected to avoid information loss or distortion as much as possible in a bid to attain reliable results [40,41]. For this purpose, Chen et al. [40] proposed a novel linguistic representation model that simultaneously factors into the generalized linguistic terms and their corresponding proportions in the context of MCGDM settings. More specifically, the proportions with respect to each generalized linguistic terms in the representation model indicates the support of each expert to the group efforts. Several theoretical extensions and real-life applications of PHFLS have demonstrated that it effectively avoid the information loss, eliminate information distortion, and facilitate the process of CW [42,43,44].

**Definition** **8** ([40])**.** *Let $S=\left\{\left.s_{i}\right|i=0,1,\cdots ,g\right\}$ be an LTS, and let $H_{S}^{k}\left(k=1,2,\cdots ,n\right)$ be n HFLTSs provided by a group of experts $E_{k}$. A PHFLTS for a linguistic variable ϑ generated by the union of $H_{S}^{k}$, denoted as $P_{H_{S}}\left(\vartheta \right)$, is a set of ordered finite proportional linguistic pairs.*

(3) $$P_{H_{S}}\left(\vartheta \right)=\left\{\left.\left(s_{i},p_{i}\right)\right|s_{i}\in S,0\leq p_{i}\leq 1,\sum_{i=0}^{g}p_{i}=1,i=0,1,\cdots ,g\right\},$$

*where $P={\left(p_{0},p_{1},\cdots ,p_{g}\right)}^{T}$ is a proportional vector and $p_{i}$ represents the possibility degree that the alternative exhibits an evaluation value $s_{i}$ given by a group of experts. Sets of $\left(s_{i},p_{i}\right)$ are named as ordered finite proportional linguistic pairs when they are ranked according to the ordered linguistic terms $s_{i}\left(i=0,1,\cdots g\right)$. For the convenience and simplicity of expression, the linguistic pairs whose proportion is equal to zero in the PHFLTS are usually omitted.*

To facilitate the application of PHFLTS, basic operational laws should be defined. Considering the defects (i.e., the calculation results exceed the limits of defined LTS and information loss) of traditional operations for LTS that usually directly conduct operations on the subscript of linguistic terms, Gou et al. [45] proposed two transformation functions *f* and $f^{-1}$ to comply the equivalent transformation between the HFLTSs and HFSs. The two transformation functions are as follows.

(4) $$\left\{\begin{array}{c} f:\left[0,g\right]\to \left[0,1\right],f\left(s_{i}\right)=\frac{Ind\left(s_{i}\right)}{g}=\frac{i}{g}=\gamma_{i},\\ f^{-1}:\left[0,1\right]\to \left[0,g\right],f^{-1}\left(\gamma_{i}\right)=s_{\gamma_{i}\times g}=s_{i}, \end{array}\right.$$

where $Ind\left(s_{i}\right)$ represents a function to derive the subscript of linguist term $s_{i}$. Based on the proposed transformation function, Yang et al. [24] proposed the basic operational laws for the computational manipulations of PHFLTS.

**Definition** **9** ([24])**.** Let $S=\left\{\left.s_{i}\right|i=0,1,\cdots ,g\right\}$ be an LTS, and let $P_{H_{S}}^{1}\left(\vartheta \right)=\left\{\left.\left(s_{i}^{1k},p_{k}^{*}\right)\right|s_{i}^{1k}\in S,0\leq p_{k}^{*}\leq 1,\sum_{k=1}^{K}p_{k}^{*}=1,i=0,1,\cdots g\right\}$ and $P_{H_{S}}^{2}\left(\vartheta \right)=\left\{\left.\left(s_{i}^{2k},p_{k}^{*}\right)\right|s_{i}^{2k}\in S,0\leq p_{k}^{*}\leq 1,\sum_{k=1}^{K}p_{k}^{*}=1,i=0,1,\cdots g\right\}$ be two PHFLTSs with the same proportional vector $P={\left(p_{1},p_{1},\cdots ,p_{K}\right)}^{T}$. Then, the following operational laws can be defined:

*(1)*  $P_{H_{S}}^{1}\left(\vartheta \right)⊕P_{H_{S}}^{2}\left(\vartheta \right)=\left\{\left.\left[f^{-1}\left(f\left(s_{i}^{1k}\right)+f\left(s_{i}^{2k}\right)-f\left(s_{i}^{1k}\right)g\left(s_{i}^{2k}\right)\right),p_{k}^{*}\right]\right|s_{i}^{1k}\in P_{H_{S}}^{1},s_{i}^{2k}\in P_{H_{S}}^{2}\right\},$
*(2)*  $P_{H_{S}}^{1}\left(\vartheta \right)⊗P_{H_{S}}^{2}\left(\vartheta \right)=\left\{\left.\left[f^{-1}\left(f\left(s_{i}^{1k}\right).f\left(s_{i}^{2k}\right)\right),p_{k}^{*}\right]\right|s_{i}^{1k}\in P_{H_{S}}^{1},s_{i}^{2k}\in P_{H_{S}}^{2}\right\},$
*(3)*  $\lambda P_{H_{S}}^{1}\left(\vartheta \right)=\left\{\left.\left[f^{-1}\left(1-{\left(1-f\left(s_{i}^{1k}\right)\right)}^{\lambda }\right),p_{k}^{*}\right]\right|s_{i}^{1k}\in P_{H_{S}}^{1}\right\},$
*(4)*  ${\left(P_{H_{S}}^{1}\left(\vartheta \right)\right)}^{\lambda }=\left\{\left.\left[f^{-1}{\left(f\left(s_{i}^{1k}\right)\right)}^{\lambda },p_{k}^{*}\right]\right|s_{i}^{1k}\in P_{H_{S}}^{1}\right\},$

*where i=0,1,⋯,g;k=1,2,⋯,K.*

## 3. Novel Comparison Laws, Distance and Entropy Measures for PHFLTS

In this section, we attempt to develop novel comparison laws as well as define a novel distance measure for PHFLTS. The entropy measure for PHFLTS is adapted from the previous work by Liu et al. [46].

**Definition** **10.** *The lower bound (min) $P_{H_{S}}^{-}$, upper bound (max) $P_{H_{S}}^{+}$, and average value of the PHFLTS $P_{H_{S}}$ are defined as:*

*(1)*  *$P_{H_{S}}^{-}=\min\left(P_{H_{S}}\right)=\min\left\{\left.r_{i}\cdot p_{i}\right|i=0,1,\cdots ,g\right\}$;*
*(2)*  *$P_{H_{S}}^{+}=\max\left(P_{H_{S}}\right)=\max\left\{\left.r_{i}\cdot p_{i}\right|i=0,1,\cdots ,g\right\}$; and*
*(3)*  *$avg\left(P_{H_{S}}\right)=\sum_{i=0}^{g}\gamma_{i}\cdot p_{i},i=0,1,\cdots ,g$ where $\gamma_{i}=\frac{Ind\left(s_{{}_{i}}\right)}{g}$, $s_{i}\in S$, and $\sum_{i=0}^{g}p_{i}=1$.*

**Definition** **11.** *The envelope of the PHFLTS, denoted as $env\left(P_{H_{S}}\right)$, is an interval value whose lower and upper bounds are determined by Definition 10. Then,*

(5) $$env\left(P_{H_{S}}\right)=\left[P_{H_{S}}^{-},P_{H_{S}}^{+}\right],$$

*which id adapted from its previous counterpart—HFLTS envelope defined by Rodríguez et al. [30].*

**Definition** **12.** *The comparison between two PHFLTSs $P_{H_{S}}^{1}\left(\vartheta \right)$ and $P_{H_{S}}^{2}\left(\vartheta \right)$ is defined based on the concept of the envelope and the average value of PHFLTS. The specific comparison rules are as follows.*

*(1)*  *If $env\left(P_{H_{S}}^{1}\left(\vartheta \right)\right)>env\left(P_{H_{S}}^{2}\left(\vartheta \right)\right)$, then $P_{H_{S}}^{1}\left(\vartheta \right)>P_{H_{S}}^{2}\left(\vartheta \right)$;*
*(2)*  *If $env\left(P_{H_{S}}^{1}\left(\vartheta \right)\right)=env\left(P_{H_{S}}^{2}\left(\vartheta \right)\right)$, then*

*(a)*  *If $\mathrm{avg}\left(P_{H_{S}}^{1}\left(\vartheta \right)\right)>avg\left(P_{H_{S}}^{2}\left(\vartheta \right)\right)$, then $P_{H_{S}}^{1}\left(\vartheta \right)>P_{H_{S}}^{2}\left(\vartheta \right)$;*
*(b)*  *If $avg\left(P_{H_{S}}^{1}\left(\vartheta \right)\right)=avg\left(P_{H_{S}}^{2}\left(\vartheta \right)\right)$, then*

*(i)*  *$P_{H_{S}}^{1}\left(\vartheta \right)>P_{H_{S}}^{2}\left(\vartheta \right),iffV\left(P_{H_{S}}^{1}\left(\vartheta \right)\right)>V\left(P_{H_{S}}^{2}\left(\vartheta \right)\right)$;*
*(ii)*  *$P_{H_{S}}^{1}\left(\vartheta \right)=P_{H_{S}}^{2}\left(\vartheta \right),iffV\left(P_{H_{S}}^{1}\left(\vartheta \right)\right)=V\left(P_{H_{S}}^{2}\left(\vartheta \right)\right)$, where $V\left(P_{H_{S}}\left(\vartheta \right)\right)=\sqrt{\sum_{i=0}^{g}{\left(\gamma_{i}-avg\left(P_{H_{S}}^{}\left(\vartheta \right)\right)\right)}^{2}.p_{i}}$ represents the variance of $P_{H_{S}}\left(\vartheta \right)$.*

It is worth noting that the comparison between arbitrary two PHFLTS envelopes is in fact comparing an interval value, which can be accomplished with reference to those defined for HFLTS envelope by Rodríguez et al. [30].

**Definition** **13.** *Let $S=\left\{\left.s_{i}\right|i=0,1,\cdots ,g\right\}$ be an LTS, and let $P_{H_{S}}^{1}$ and $P_{H_{S}}^{2}$ be two PHFLTSs. The definition of the distance measure between them could be put forward as follows.*

(6) $$d\left(P_{H_{S}}^{1},P_{H_{S}}^{2}\right)=\frac{\left|P_{H_{S}}^{1-}-P_{H_{S}}^{2-}\right|+\left|P_{H_{S}}^{1+}-P_{H_{S}}^{2+}\right|+\left|avg\left(P_{H_{S}}^{1}\right)-avg\left(P_{H_{S}}^{2}\right)\right|+\left|V\left(P_{H_{S}}^{1}\right)-V\left(P_{H_{S}}^{2}\right)\right|}{4},$$

*where the $P_{H_{S}}^{-}$, $P_{H_{S}}^{+}$, and $avg\left(P_{H_{S}}\right)$ have been defined in Definition 10.*

The defined distance measure for PHFLTSs obviously satisfies the following axiomatic requirements:

(a) $0\leq d\left(P_{H_{S}}^{1},P_{H_{S}}^{2}\right)\leq 1$;
(b) $d\left(P_{H_{S}}^{1},P_{H_{S}}^{2}\right)=0$, if and only if $P_{H_{S}}^{1}=P_{H_{S}}^{2}$; and
(c) $d\left(P_{H_{S}}^{1},P_{H_{S}}^{2}\right)=d\left(P_{H_{S}}^{2},P_{H_{S}}^{1}\right)$.

**Proof.**  The proof of (a) and (c) is obvious, and thus omitted here. We only need to prove that the proposed distance measure satisfies (b).

Firstly, we have

$$d\left(P_{H_{S}}^{1},P_{H_{S}}^{2}\right)=\frac{1}{4}\left(\left|P_{H_{S}}^{1-}-P_{H_{S}}^{2-}\right|+\left|P_{H_{S}}^{1+}-P_{H_{S}}^{2+}\right|+\left|avg\left(P_{H_{S}}^{1}\right)-avg\left(P_{H_{S}}^{2}\right)\right|+\left|V\left(P_{H_{S}}^{1}\right)-V\left(P_{H_{S}}^{2}\right)\right|\right),$$

and

$$0\leq \left|P_{H_{S}}^{1-}-P_{H_{S}}^{2-}\right|\leq 1,0\leq \left|P_{H_{S}}^{1+}-P_{H_{S}}^{2+}\right|\leq 1,0\leq \left|avg\left(P_{H_{S}}^{1}\right)-avg\left(P_{H_{S}}^{2}\right)\right|\leq 1.$$

It is evident that $0\leq \left|V\left(P_{H_{S}}^{1}\right)-V\left(P_{H_{S}}^{2}\right)\right|\leq 1$, $d\left(P_{H_{S}}^{1},P_{H_{S}}^{2}\right)=0$ if and only if the conditions that $\left|P_{H_{S}}^{1-}-P_{H_{S}}^{2-}\right|=0$, and $\left|V\left(P_{H_{S}}^{1}\right)-V\left(P_{H_{S}}^{2}\right)\right|=0$, are satisfied simultaneously, that is, $P_{H_{S}}^{1-}=P_{H_{S}}^{2-}$, $P_{H_{S}}^{1+}=P_{H_{S}}^{2+}$, $avg\left(P_{H_{S}}^{1}\right)=avg\left(P_{H_{S}}^{2}\right)$, and $\left|V\left(P_{H_{S}}^{1}\right)-V\left(P_{H_{S}}^{2}\right)\right|=0$. Then, based on the Definition 12, the above conditions could be satisfied only when $P_{H_{S}}^{1}=P_{H_{S}}^{2}$. Therefore, $d\left(P_{H_{S}}^{1},P_{H_{S}}^{2}\right)=0$, if and only if $P_{H_{S}}^{1}=P_{H_{S}}^{2}$.

The proof is completed. □

Given the proposed distance measure, we can define the similarity measure for PHFLTS based on Zadeh’s negation as follows.

(7) $$\rho \left(P_{H_{S}}^{1},P_{H_{S}}^{2}\right)=1-\frac{\left|P_{H_{S}}^{1-}-P_{H_{S}}^{2-}\right|+\left|P_{H_{S}}^{1+}-P_{H_{S}}^{2+}\right|+\left|avg\left(P_{H_{S}}^{1}\right)-avg\left(P_{H_{S}}^{2}\right)\right|+\left|V\left(P_{H_{S}}^{1}\right)-V\left(P_{H_{S}}^{2}\right)\right|}{4}.$$

Similarly, the defined similarity measure satisfies the following basic axiomatic requirements:

(a) $0\leq \rho \left(P_{H_{S}}^{1},P_{H_{S}}^{2}\right)\leq 1$;
(b) $\rho \left(P_{H_{S}}^{1},P_{H_{S}}^{2}\right)=1$, if and only if $P_{H_{S}}^{1}=P_{H_{S}}^{2}$; and
(c) $\rho \left(P_{H_{S}}^{1},P_{H_{S}}^{2}\right)=\rho \left(P_{H_{S}}^{2},P_{H_{S}}^{1}\right)$.

The proofs are similar to those of the distance measure for PHFLTS, and thus they are omitted here.

The entropy measure is an important tool to express the mathematical values of the fuzziness of PHFLTS. We adapt the following entropy measures of PHFLTSs from those defined by Wei et al. [39] for HFLTSs and those defined for probabilistic linguistic term set by Liu et al. [46].

**Definition** **14.** *Let $S=\left\{\left.s_{i}\right|i=0,1,\cdots ,g\right\}$ be an LTS, and let $P_{H_{S}}\left(\vartheta \right)$ be a PHFLTS. Then, the entropy measure for PHFLTSs is defined as follows.*

*(1)*  *$E_{1}\left(P_{H_{S}}\right)=\sum_{i=0}^{g}4p_{i}\left[\gamma_{i}.\left(1-\gamma_{i}\right)\right]$;*
*(2)*  *$E_{2}\left(P_{H_{S}}\right)=\frac{1}{\ln2}\sum_{i=0}^{g}p_{i}.\left[\gamma_{i}.\left|\ln\gamma_{i}\right|+\left(1-\gamma_{i}\right).\left|ln\left(1-\gamma_{i}\right)\right|\right]$; and*
*(3)*  *$E_{3}\left(P_{H_{S}}\right)=\frac{1}{\sqrt{e}-1}\sum_{i=0}^{g}p_{i}.\left[\gamma_{i}.e^{1-\gamma_{i}}+\left(1-\gamma_{i}\right).e^{\gamma_{i}}-1\right]$.*

*Especially, we adopt the convention ln0=0.*

**Proposition** **1.** *The entropy measure defined in Definition 14 satisfies the following properties:*

*(1)*  *$E_{i}\left(P_{H_{S}}\right)=0,i=1,2,3,iffP_{H_{S}}=\left\{\left(s_{0},1\right)\right\},P_{H_{S}}=\left\{\left(s_{g},1\right)\right\},orP_{H_{S}}=\left\{\left(s_{0},p\right)\left(s_{g},1-p\right)\right\}$,*
*(2)*  *EiPHS=1,i=1,2,3,iffPHS=sgg22,1.*

The proofs of Proposition 1 are similar to those of Wei et al. [39] and Liu et al. [46] and, therefore, are omitted here. Furthermore, one can refer to the works of Tian et al. [47] and Gou et al. [48] for more definitions of information measures such as cross-entropy, relative entropy, and hesitant entropy. The proposed entropy measure for PHFLTS entails the advantages of superior interpretability and applicability. In addition, it preserves the ability to measure the information contained in PHFLTSs and assure the reliability of the derived weights, as discussed in the subsequent section.

## 4. Integrated PHFL-TOPSIS Model for HazMat Transportation Alternative Evaluation

**Figure 1:**
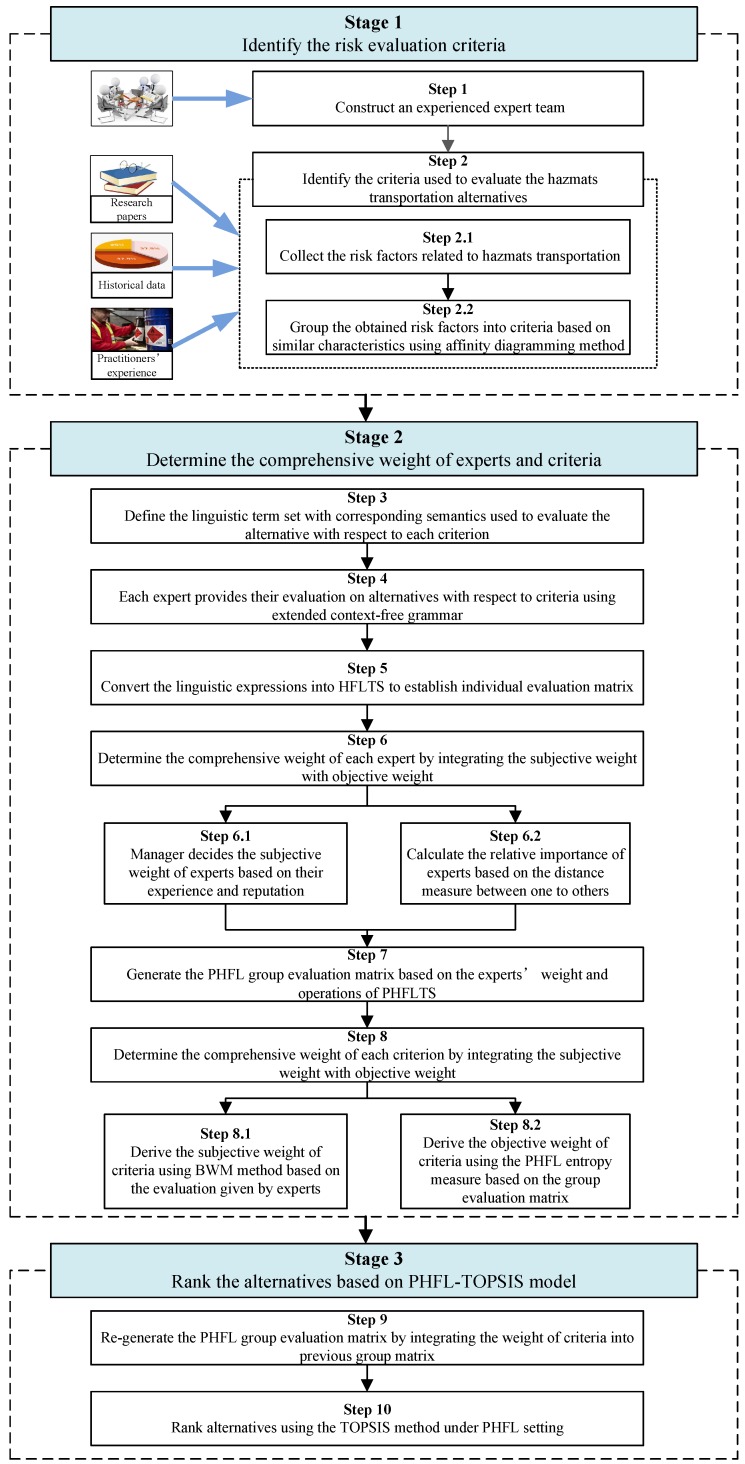
The flowchart of the proposed PHFL-TOPSIS model for alternative evaluation.

The HazMat transportation accompanied with various risks and may lead to catastrophic disasters to human life, social economic, and natural environment once an accident happened. An alternative evaluation and selection model based on risk evaluation must, therefore, be developed to identify the most appropriate alternative for manufacturing enterprises. The PHFLTS is an effective information representation model to take into account the qualitative linguistic evaluations as well as their corresponding proportions when the opinions of a group of experts are gathered. TOPSIS is a widely used MCDM method and has been successfully used in many fields [16,17,44]. Therefore, in this section, we propose an extended TOPSIS model with PHFL information to deal with the alternative evaluation and selection problem of HazMat transportation. The integrated PHFL-TOPSIS model is mainly composed by three stages: identifying the risk criteria for alternative evaluation, determining the comprehensive weight information of risk criteria and experts, and ranking the alternative for HazMat transportation. The flowchart of the proposed integrated PHFL-TOPSIS model is as shown in Figure 1. More details on each step involved in the model are elaborated in the subsequent subsections.

### 4.1. Identification of the Risk Evaluation Criteria

*Step 1. Construct an Experienced Expert Team.*

The core input of proposed method is the evaluation information offered by the invited experts, it is an important preparation work to establish an experience expert team whose members are of rich experience, knowledge, and reputation in the field of HazMat transportation. We denote $E=\left\{e_{1},e_{2},\cdots ,e_{T}\right\}$ as the set of experts, where *T* represents the number of experts participated in the decision-making process.

*Step 2. Identify the risk evaluation criteria.*

To evaluate and select the best HazMat transportation alternative, we need first to determine the evaluation criteria used during the decision-making process. We denote the candidate HazMat transportation alternatives as $P=\left(p_{1},p_{2},\cdots ,p_{i},\cdots p_{m}\right)$, where *m* represents the number of alternatives. There are many risk factors that have close relationship with the HazMat transportation accidents. Therefore, collecting those risk factors is necessary to determine the risk evaluation criteria, which are sourced from existing research papers in the field, collected and stored historical data of HazMat accidents, regulation files for HazMat transportation, and the experience provided by the first-line practitioners. Some of the collected risk factors may be of similar characteristics to each other, and thus it is necessary to group them into clusters based on their characteristics. Many methods, such as the cause and effect analysis diagram or fishbone diagram, and affinity diagramming, have been proposed to cluster the items. Among them, affinity diagramming is a widely used method for this purpose [49]. This method not only improves the efficiency of clustering process but also facilitates the adjustment process. Subsequently, each cluster is given a name that can describe the risk factors included in it. These clusters can be used as the criteria for risk evaluation. We denote the risk evaluation criteria vector as $U=\left(u_{1},u_{2},\cdots ,u_{j},\cdots ,u_{n}\right)$, where *n* represents the number of criteria.

### 4.2. Determination of the Comprehensive Weight Information of Experts and Criteria

The weights of experts and criteria have direct influence on the evaluation results in MCGDM contexts. In this sense, it is important to consider the weight of experts and criteria simultaneously. Furthermore, to obtain more reliable results, the weight information should not only reflect the subjective preference of experts but also the objective information contained in the evaluation information. For this purpose, we propose two comprehensive weight assignment models, which integrate the subjective preference with objective information to determine the weights of experts and criteria based on the distance and entropy measures of PHFLTS, respectively. The benefits of using the distance and entropy measures for PHFLTS are two-folds. On the one hand, the weights derived from these two measures are objective, and, on the other hand, the evaluation information could be taken full advantage of. Therefore, the weights determined by these two methods are reliable. It is worth noting that other methods such as adaptive-consensus-based method can also be used to derive the weights as per the different requirements under various settings.

*Step 3. Define the LTS with corresponding semantics.*

Considering the fuzziness and uncertainty contained in the process of risk evaluation, the evaluation and selection of HazMat transportation alternatives can be regarded as a qualitative MCGDM problem. Therefore, it is necessary to define the LTS as well as their semantics used to assess the alternatives with respect to the criteria. The granularity of the LTS can be neither too big nor too small [50,51,52]. If the granularity is too big, then the difference between the adjacent linguistic terms will be difficult to identify; however, if it is too small, the accuracy of results will decrease. Considering both aspects, we define the LTS used in this study with seven granularities. The specific semantic and representation is as follows.

$$S=\left\{\begin{array}{c} s_{0}:\;\;very\;\;low\;\;\left(VL\right),\;s_{1}:\;\;moderate\;\;low\;\;\left(ML\right),s_{2}:\;\;low\;\;\left(L\right),\;s_{3}:moderate\;\;\left(M\right),\\ s_{4}:\;high\;\;\left(H\right),\;s_{5}:\;\;moderate\;\;high\;\;\left(MH\right),\;s_{6}:\;very\;\;high\;\;\left(VH\right) \end{array}\right\}.$$

Besides, the LTS with five or nine granularities also could be used when the experts are either not so familiar or, on the contrary, very familiar with the problem. The determination of granularity is closely related to the complexity and accuracy of the background of real-world applications.

*Step 4. Evaluate the alternative using extended context-free grammar.*

Considering the heterogeneity of each expert in the aspects of experience and knowledge, experts are permitted to use thecontext-free grammar shown in Definition 2 to express their evaluation information. For example, one expert may think that the risk of alternative $p_{i}$ with respect to criterion $u_{j}$ is “*between M and VH*”.

*Step 5. Convert the linguistic expressions into HFLTS.*

For subsequent calculations, the linguistic expressions provided by experts need to be converted into HFLTSs. The transformation function $E_{G_{H}}$ defined in Definition 3 is used to reach the action. Then, HFLTS-represented evaluation matrix $V_{t}={\left[H_{S_{ij}}^{t}\right]}_{m\times n}$ could be established corresponding to each expert, where $H_{S_{ij}}^{t}$ is an HFLTS and means the evaluation of alternative $p_{i}$ with respect to criterion $u_{i}$ provided by expert $e_{i}$.

*Step 6. Determine the comprehensive weight of experts.*

The weights of experts represent their discourse power and have direct impact on the decision results. The expert weights should show their status in the field, the preference of enterprise manager, and their professional knowledge, and thus it must contain both of the subjective part and objective part.

*Step 6.1. Determine the subjective weight of experts.*

The subjective weights of experts in this study are determined by the manager based on their experience and reputation. If the experts have worked with the related problem for many years or have dealt with a significant amount of related projects and have good reputations in the related field, they should be given larger weights. Otherwise if the experts are less experienced with moderate reputation, lower weights should be assigned to them. We denote the subjective weight of experts as $W^{S}=\left(w_{1}^{s},w_{2}^{s},\cdots ,w_{t}^{s}\right)$.

*Step 6.2. Determine the objective weight of experts.*

The objective weights of experts is determined by measuring the distance between their individual evaluations to the rest. The distance measure for HFLTS in Definition 7 is used to achieve this purpose. We denote the objective weights of experts as $W^{O}=\left(w_{1}^{o},w_{2}^{o},\cdots ,w_{t}^{o}\right)$. The specific process of determining the objective weight of experts is shown below.

The expert weights can be obtained by measuring the consistency degree among experts each other. Further, the consistency degree between experts can be represented by the similarity between their evaluation on each alternative with respect to each criterion. Assume that there are two experts $e_{k}$ and $e_{l}$, the evaluation information of alternative $p_{i}$ under the criterion $u_{j}$ given by them is represented by $H_{S_{ij}}^{k}$ and $H_{S_{ij}}^{l}$, respectively. Thus, the similarity between the two experts can be calculated using the distance and similarity measures for HFLTS based on Definition 7.

*Step 6.2.1. To calculate the distance of alternative $p_{i}$, denoted as $D_{i}^{kl}$, between experts $e_{k}$ and $e_{l}$.*

(8) $$D_{i}^{kl}=\frac{1}{n}\sum_{j=1}^{n}d\left(H_{S_{ij}}^{k},H_{S_{ij}}^{l}\right),$$

where *n* represents the number of criteria.

*Step 6.2.2. To calculate the similarity of alternative $p_{i}$, denoted as $CD_{i}^{kl}$*, between experts $e_{k}$ and $e_{l}$

(9) $$CD_{i}^{kl}=1-D_{i}^{kl}=1-\frac{1}{n}\sum_{j=1}^{n}d\left(H_{S_{ij}}^{k},H_{S_{ij}}^{l}\right).$$

*Step 6.2.3. A consistency degree matrix ${\mathrm{CD}}_{i}$ can be constructed to show the consistency degree* between any two experts as follows.

$${\mathrm{CD}}_{i}=\left[\begin{array}{cccc} 1 & CD_{i}^{12} & \cdots & CD_{i}^{1t}\\ CD_{i}^{21} & 1 & \cdots & CD_{i}^{2t}\\ \vdots & \vdots & \vdots & \vdots \\ CD_{i}^{t1} & CD_{i}^{t2} & \cdots & 1 \end{array}\right]$$

where *t* represents the number of experts and $CD_{i}^{kl}=CD_{i}^{lk},CD_{i}^{tt}=1,i=1,2,\cdots ,m$.

*Step 6.2.4. The averaging consistency degree of expert $e_{k}$ corresponding to alternative $p_{i}$* could be represented by

(10) $$AD_{i}^{k}=\frac{1}{t-1}\sum_{h=1,h\neq k}^{t}CD_{i}^{kh}.$$

*Step 6.2.5. The relative consistency degree of expert $e_{k}$ to others corresponding to alternative $p_{i}$* could be represented by

(11) $$RD_{i}^{k}=\frac{AD_{i}^{k}}{\sum_{k=1}^{t}AD_{i}^{k}}.$$

*Step 6.2.6. For all the m alternatives, the sum of relative consistency degree of expert $e_{k}$* to others is

(12) $$SD^{k}=\sum_{i=1}^{m}RD_{i}^{k}.$$

*Step 6.2.7. The objective weight of each expert can be obtained by normalizing the SD.*

(13) $$w_{k}^{o}=\frac{SD^{k}}{\sum_{k=1}^{t}SD}.$$

Finally, the comprehensive weight vector of experts is $W=\left(w_{1},w_{2},\cdots ,w_{t}\right)$, where $w_{k}=\alpha w_{k}^{S}+\left(1-\alpha \right)w_{k}^{O},k=1,2,\cdots ,t$ is an integration of the subjective and objective weights.

*Step 7. Generate the PHFL group evaluation matrix.*

Based on the obtained comprehensive weight of experts, the HFLTS-represented evaluation matrix given by each expert could be integrated to generate the PHFL group evaluation matrix using the operations of PHFLTS in Definition 9, which is

$$R={\left[P_{H_{S_{ij}}}\right]}_{m\times n},$$

where $P_{H_{S_{ij}}}$ is a PHFLTS that represents the group evaluation of alternative $p_{i}$ with respect to the criterion $u_{i}$.

*Step 8. Determine the comprehensive weight of criteria.*

Similar to the process of expert weight determination, the criteria weights should be a reflection of the subjective reference of experts and the objective information contained in the evaluation simultaneously.

*Step 8.1. Derive the subjective weight of criteria using BWM.*

Many methods such as AHP, Delphi, and directly scoring have been proposed to derive the subjective weights and have been applied in MCDM/MCGDM [3,18]. The BWM proposed by Rezaei [53] is an effective subjective weight determination model based on pair-wise comparison among criteria that is similar to AHP. However, BWM requires fewer comparisons, which in turn decreases the complexity and increases the consistency of results compared to the AHP, and it can assure the consistency and avoid the arbitrariness compared to Delphi and directly scoring methods. For more detailed information about BWM, readers are suggested to refer to the work of Rezaei [53]. We denote the obtained subjective weight of criteria based on the evaluation of experts as $\psi^{S}=\left(\varphi_{1}^{S},\varphi_{2}^{S},\cdots ,\varphi_{n}^{S}\right)$.

*Step 8.2. Derive the objective weight of criteria.*

The objective weights of criteria are usually calculated by measuring the information contained in the evaluation results. Entropy measure is an effective way to measure the information volume and uncertainty, and thus the entropy measure for PHFLTS proposed in Definition 14 is used here. Two sub-steps are included in the process.

*Step 8.2.1. Calculate the entropy of criterion $u_{i}$ under different alternatives.*

(14) $$E_{j}=\frac{1}{m}\sum_{i=1}^{m}E\left(P_{H_{S}}^{ij}\right),j=1,2,\cdots ,n,$$

where $P_{H_{S}}^{ij}$ is a PHFLTS and represents the evaluation of criterion $u_{i}$ under alternative $p_{i}$, $E\left(P_{H_{S}}^{ij}\right)$ can be any entropy measure for PHFLTS proposed in Definition 14.

*Step 8.2.2. Calculate the objective weight of criterion based on the obtained entropy information.*

(15) $$\varphi_{j}^{o}=\frac{1-E_{j}}{\sum_{j=1}^{n}\left(1-E_{j}\right)}=\frac{1-E_{j}}{n-\sum_{j=1}^{n}E_{j}}.$$

Finally, the comprehensive weight vector of criteria is $\psi =\left(\varphi_{1},\varphi_{2},\cdots ,\varphi_{n}\right)$, where $\varphi_{j}=\beta \varphi_{j}^{S}+\left(1-\beta \right)\varphi_{j}^{O},j=1,2,\cdots ,n$ is an integration of subjective and objective weights obtained in the previous.

Based on the above analysis, the detailed and comprehensive procedures to determine the comprehensive weight of experts and criteria could be simply depicted below to facilitate their algorithmic implementation and computational manipulation.

**Algorithm 1:** Determine the comprehensive weights of experts and criteria.**Inputs:***S*, $E=\left\{e_{1},e_{2},\cdots ,e_{T}\right\}$, $P=\left(p_{1},p_{2},\cdots ,p_{i},\cdots p_{m}\right)$, $U=\left(u_{1},u_{2},\cdots ,u_{j},\cdots ,u_{n}\right)$ **Outputs:**$W=\left(w_{1},w_{2},\cdots ,w_{t}\right)$, $\psi =\left(\varphi_{1},\varphi_{2},\cdots ,\varphi_{n}\right)$ **Step 1.** Define the LTS with corresponding semantics, $S=\left\{\left.s_{i}\right|i=0,1,\cdots ,g\right\}$ **Step 2.** Evaluate the alternative using extended context-free grammar. **Step 3.** Convert the linguistic expressions into HFLTS to establish individual evaluation matrix $V_{t}={\left[H_{S_{ij}}^{t}\right]}_{m\times n}$ **Step 4.** Determine the comprehensive weight of experts, $W=\left(w_{1},w_{2},\cdots ,w_{t}\right)$ **Step 4.1.** Determine the subjective weight of experts $W^{S}=\left(w_{1}^{s},w_{2}^{s},\cdots ,w_{t}^{s}\right)$ by managers  **Step 4.2.** Determine the objective weight of experts $W^{O}=\left(w_{1}^{o},w_{2}^{o},\cdots ,w_{t}^{o}\right)$
  **Step 4.2.1.** Calculate the distance of alternative $p_{i}$, denoted as $D_{i}^{kl}$, between experts $e_{k}$ and $e_{l}$, $D_{i}^{kl}=\frac{1}{n}\sum_{j=1}^{n}d\left(H_{S_{ij}}^{k},H_{S_{ij}}^{l}\right)$
**  Step 4.2.2.** Calculate the similarity of alternative $p_{i}$, denoted as $CD_{i}^{kl}$, between experts $e_{k}$ and $e_{l}$, $CD_{i}^{kl}=1-D_{i}^{kl}=1-\frac{1}{n}\sum_{j=1}^{n}d\left(H_{S_{ij}}^{k},H_{S_{ij}}^{l}\right)$  **Step 4.2.3.** Construct a consistency degree matrix of alternative $p_{i}$ among experts ${\mathrm{CD}}_{i}={\left[CD_{i}^{kl}\right]}_{t\times t}$  **Step 4.2.4.** Calculate the averaging consistency degree of expert $e_{k}$ corresponding to alternative $p_{i}$, $AD_{i}^{k}=\frac{1}{t-1}\sum_{h=1,h\neq k}^{t}CD_{i}^{kh}$  **Step 4.2.5.** Calculate the relative consistency degree of expert $e_{k}$ to others corresponding to alternative $p_{i}$, $RD_{i}^{k}=\frac{AD_{i}^{k}}{\sum_{k=1}^{t}AD_{i}^{k}}$
  **Step 4.2.6.** Calculate the sum of relative consistency degree of all alternatives to expert $e_{k}$, $SD^{k}=\sum_{i=1}^{m}RD_{i}^{k}$
  **Step 4.2.7.** Calculate the objective weight of each expert by normalizing the SD, $w_{k}^{o}=\frac{SD^{k}}{\sum_{k=1}^{t}SD}$ **Step 4.3.** Calculate the comprehensive weight of experts, that is, $w_{k}=\alpha w_{k}^{S}+\left(1-\alpha \right)w_{k}^{O},k=1,2,\cdots ,t$ **Step 5.** Generate the PHFL group evaluation matrix $R={\left[P_{H_{S_{ij}}}\right]}_{m\times n}$ based on the obtained weight of experts **Step 6.** Determine the comprehensive weight of criteria, $\psi =\left(\varphi_{1},\varphi_{2},\cdots ,\varphi_{n}\right)$ **Step 6.1.** Derive the subjective weight of criteria using best to worst method (BWM), $\psi^{S}=\left(\varphi_{1}^{S},\varphi_{2}^{S},\cdots ,\varphi_{n}^{S}\right)$ **Step 6.2.** Derive the objective weight of criteria, $\psi^{S}=\left(\varphi_{1}^{S},\varphi_{2}^{S},\cdots ,\varphi_{n}^{S}\right)$
  **Step 6.2.1.** Calculate the entropy of criterion $u_{i}$ under different alternatives, $E_{j}=\frac{1}{m}\sum_{i=1}^{m}E\left(P_{H_{S}}^{ij}\right),j=1,2,\cdots ,n$  **Step 6.2.2.** Calculate the objective weight of criterion $u_{i}$ based on the obtained entropy information, $\varphi_{j}^{o}=\frac{1-E_{j}}{\sum_{j=1}^{n}\left(1-E_{j}\right)}=\frac{1-E_{j}}{n-\sum_{j=1}^{n}E_{j}}$
 **Step 6.3.** Calculate the comprehensive weight of criteria, that is, $\varphi_{j}=\beta \varphi_{j}^{S}+\left(1-\beta \right)\varphi_{j}^{O},j=1,2,\cdots ,n$
**End**

### 4.3. Rank the Alternative Based on Extended PHFL-TOPSIS Method

*Step 9. Regenerate the PHFL group evaluation matrix*

After the weights of experts and criteria are determined comprehensively, the group evaluation matrix could be re-generated based on the comprehensive weights of experts and criteria. Then, the re-generated group evaluation matrix represented by PHFLTS, denoted as $\bar{R}$, is

$$\bar{R}={\left[{\bar{P}}_{H_{S_{ij}}}\right]}_{m\times n},$$

where ${\bar{P}}_{H_{S_{ij}}}$ is a PHFLTS that represents the group evaluation of alternative $p_{i}$ with respect to the criterion $u_{j}$ based on the comprehensive weights of experts $w_{t}$ and criteria $\varphi_{j}$.

*Step 10. Rank alternatives using PHFL-TOPSIS method.*

With the derivation of the group evaluation matrix, the PHFL-TOPSIS method is developed in the sequel for the final ranking of alternatives.

*Step 10.1. Define the positive solutions $P_{H_{S}}^{j+}$ and negative solutions $P_{H_{S}}^{j-}$.*

The positive and negative solutions should be defined based on the characteristics of criteria. Criteria can be normally group into two types, namely, cost type ($\Omega_{c}$) and benefit type ($\Omega_{b}$). For the criteria that belong to cost type, the smaller is the value, the better and vice versa. For the criteria that belong to benefit type, the bigger is the value, the better and vice versa. Therefore,

(16) $$P_{H_{S}}^{j+}=\left\{\begin{array}{c} \max_{1\leq i\leq m}P_{{}^{H_{S}}}^{ij},j\in \Omega_{b}\\ \min_{1\leq i\leq m}P_{H_{S}}^{ij},j\in \Omega_{c} \end{array}\right.,P_{H_{S}}^{j-}=\left\{\begin{array}{c} \max_{1\leq i\leq m}P_{{}^{H_{S}}}^{ij},j\in \Omega_{c}\\ \min_{1\leq i\leq m}P_{H_{S}}^{ij},j\in \Omega_{b} \end{array}\right.,j=1,2,\cdots ,n$$

Especially, the comparison method for PHFLTS proposed in Definition 12 is used here.

*Step 10.2. Calculate the positive distance $D_{i}^{+}$ between the evaluation value ${\bar{P}}_{H_{S}}^{ij}$ and $P_{H_{S}}^{j+}$ as well as the negative distance $D_{i}^{-}$ between the evaluation value ${\bar{P}}_{H_{S}}^{ij}$ and $P_{H_{S}}^{j-}$*

(17) $$D_{i}^{+}=\sum_{j=1}^{n}d\left({\bar{P}}_{H_{S}}^{ij},P_{H_{S}}^{j+}\right),D_{i}^{-}=\sum_{j=1}^{n}d\left({\bar{P}}_{H_{S}}^{ij},P_{H_{S}}^{j-}\right),i=1,2,\cdots ,m$$

Especially, the distance measure for PHFLTS proposed in Definition 13 is used here.

Step 10.3. Calculate the ranking index $C_{i}^{*}$

(18) $$C_{i}^{*}=\frac{D_{i}^{-}}{D_{i}^{+}+D_{i}^{-}}$$

Finally, all alternatives can be ranked according to $C_{i}^{*}$. Obviously, $C_{i}^{*}\in \left[0,1\right]$ and the bigger the $C_{i}^{*}$ is, the better the alternative is.

## 5. Case Study and Comparison Analysis

### 5.1. An Illustrative Example

A HazMat manufacturing company in Sichuan Province, China has a batch of HazMat with explosivity and ignitability that needs to be transported from Sichuan to Guizhou. The company has more than one thousand employees and has a group of technological talents who are equipped with corresponding professional skills. The main business scopes and competitive advantages of the company are manufacturing and selling the blasting products and providing blasting technology services. In light of the high risk and professional equipment requirements, the company decides to outsource the business to professional HazMat transportation. After the tender and preliminary screening of the alternative companies, five companies become the potential cooperator. Five transportation alternatives, denoted as $\left\{p_{1},p_{2},p_{3},p_{4},p_{5}\right\}$, are provided by the transportation company. The five transportation alternatives have different advantages with respect to various evaluation criteria. To choose reliably the best transportation alternative with lowest risk level, the integrated MCGDM approach based on the PHFL-TOPSIS proposed was applied to solve the multiple criteria HazMat transportation alternative evaluation and selection problem. The case was selected because it matches perfectly with our research item, that is, multiple risk factors are concerned in the HazMat transportation process, and multiple transportation alternatives are ready to be selected. Therefore, the feasibility and effectiveness of the proposed method could be demonstrated by the application. An experienced expert team in the field should be constructed first, and the rules of conducting evaluation are illustrated to each expert for collecting reliable initial evaluation information. Specifically, the context-free grammar is explained to each expert for facilitating the evaluation information expression. The specific evaluation and selection processes are detailed in the sequel.

**Step 1.** Construct an experienced team. For making a reliable decision, five experts denoted as $\left\{e_{1},e_{2},e_{3},e_{4},e_{5}\right\}$ in the field of HazMat transportation are invited to construct a decision-making team for choosing the best transportation alternative.

**Figure 2:**
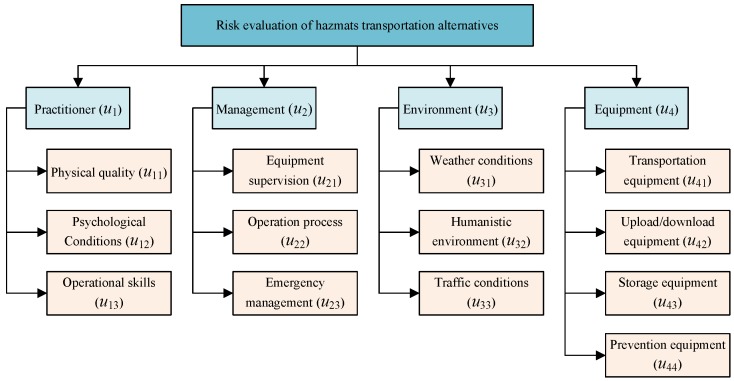
Risk evaluation criteria of HazMat transportation alternatives.

**Step 2.** Identify the criteria used to evaluate the alternatives. To evaluate the risk of each alternative, the risk factors associated with HazMat transportation need to be identified first. By means of searching related research papers [9,10,54,55,56,57,58,59,60,61], historical transportation and accidents data, and interviewing experienced practitioners who have worked in the first line for over ten years, a hierarchical transportation risk evaluation index system, which has four first-level criteria—Human ($u_{1}$), Management ($u_{2}$), Environment ($u_{3}$), Equipment ($u_{4}$)—with 13 risk indictors is established, as shown in Figure 2.

*Explanation of the risk evaluation index system*

- Practitioners ($u_{1}$)
  Practitioners are the direct risk factors that related to the HazMat transportation accidents. Three risk indicators related to practitioners are identified.
  (1) Physical quality ($u_{11}$). This risk indicator mainly includes the age and body quality of the practitioners.
  (2) Psychological conditions ($u_{12}$). This risk indicator relates to the safety awareness, emotional adjustment ability and compression ability under high-risky working environment.
  (3) Operational skills ($u_{13}$). The indicator means the professional skills of the practitioners when operating the equipment and HazMat.
- Management ($u_{2}$)
  Management is an indirect risk factors that could affect the HazMat transportation accidents. Three indicators belong to Management criteria.
  (1) Equipment supervision ($u_{21}$). This indicator relates to the procurement, audit, and maintenance of transportation and operation equipment.
  (2) Operation process ($u_{22}$). This indicator relates to the regulatory operation methods, operation sequence of the related equipment and HazMat.
  (3) Emergency management ($u_{23}$). It includes the development and perfection of emergency plan before accidents as well as the response and execution of emergency plan when accidents happen.
- Environment ($u_{3}$)
  Environment is also an indirect risk factors to transportation accidents. Three risk indicators are included in it.
  (1) Weather conditions ($u_{31}$). Weather conditions may influence the characteristics of HazMat, equipment and practitioners, therefore extremely bad weather such as heavy rain, snow, and fog should be avoided when transporting the HazMat.
  (2) Humanistic environment ($u_{32}$). The social conditions, such as population density, social order, and customs have close relationship with the probability and severity degree of transportation accidents.
  (3) Traffic conditions ($u_{33}$). The terrain, geology and unobstructed degree along the transportation road also have impact on the transportation accidents.
- Equipment ($u_{4}$)
  Equipment is the supporter of HazMat transportation and is directly related to the transportation risk. Four risk indicators are identified in this criterion.
  (1) Transportation equipment ($u_{41}$). This usually means transportation vehicles equipped with special containers and it is the most related indicator to transportation accidents.
  (2) Upload/download equipment ($u_{42}$). Specialized forklift and crane should be equipped to operate the HazMat before and after the transportation.
  (3) Storage equipment ($u_{43}$). The HazMat might not be able to be directly transported to the destination; it may need some storage equipment and places during the temporary transfer.
  (4) Prevention equipment ($u_{44}$). Isolation equipment, emergency handling device, and alternative equipment are needed to protect the practitioners and to prevent accidents from expanding.

Although 13 risk indicators are identified, we do not have necessarily to use them directly as the criteria when evaluating the transportation alternatives for three main reasons. Firstly, while the reliability of evaluation may be enhanced with larger sample size and quantity of risk factors, too many criteria would elevate the cost of finance, data collection, analysis and documentation [62]. Secondly, too many indicators not only increases the workload but also decreases the consistency of evaluation information, which then decreases the reliability of results. Thirdly, all the risk factors are derived by the expert team, and they are very familiar with the risk indicators contained in each first-level criteria. Therefore, even if we used the first-level criteria, it would not lead to the uncompleted evaluation. To this end, we choose Human ($u_{1}$), Management ($u_{2}$), Environment ($u_{3}$), and Equipment ($u_{4}$) as four criteria for evaluating the HazMat transportation alternatives. The advantage of using these four criteria is to reduce the complexity and workload of experts evaluation while at the same time not neglecting any identified risk factors. In addition, if we cluster the risk factors from different perspectives, then different criteria may be used. However, all the risk factors should be contained into the evaluation criteria for reliable results.

**Step 3.** Define the LTS with corresponding semantics. As stated in Section 4.2, the LTS used in this study is

$$S=\left\{\begin{array}{c} s_{0}:\;\;very\;\;low\;\;\left(VL\right),\;s_{1}:\;\;moderate\;\;low\;\;\left(ML\right),s_{2}:\;\;low\;\;\left(L\right),\;s_{3}:moderate\;\;\left(M\right),\\ s_{4}:\;high\;\;\left(H\right),\;s_{5}:\;\;moderate\;\;high\;\;\left(MH\right),\;s_{6}:\;very\;\;high\;\;\left(VH\right) \end{array}\right\}.$$

**Step 4.** Evaluate the alternative using extended context-free grammar. Each expert is different from experience, knowledge, and thinking habits, thus different linguistic expressions are provided according to their preference. The evaluation information provided by experts is shown in Table 1.

**Step 5.** Convert the linguistic expressions into HFLTS. For subsequent calculation, the linguistic expressions in Table 1 should be converted into HFLTSs. The transformation function $E_{G_{H}}$ in Definition 4 is used here. Then, HFLTS-represented individual evaluation matrices could be established. For example, the matrix corresponding to expert $e_{1}$ is as follows. The completed HFLTS-represented individual evaluation matrices can be referred to in Appendix A.

$$V_{1}=\left[\begin{array}{cccc} \left\{s_{3},s_{4},s_{5}\right\} & \left\{s_{3},s_{4},s_{5},s_{6}\right\} & \left\{s_{3}\right\} & \left\{s_{3},s_{4},s_{5}\right\}\\ \left\{s_{6}\right\} & \left\{s_{4}\right\} & \left\{s_{4},s_{5},s_{6}\right\} & \left\{s_{0},s_{1},s_{2},s_{3}\right\}\\ \left\{s_{0},s_{1},s_{2}\right\} & \left\{s_{6}\right\} & \left\{s_{1},s_{2},s_{3}\right\} & \left\{s_{6}\right\}\\ \left\{s_{5},s_{6}\right\} & \left\{s_{3},s_{4},s_{5},s_{6}\right\} & \left\{s_{4},s_{5},s_{6}\right\} & \left\{s_{0},s_{1},s_{2}\right\}\\ \left\{s_{1},s_{2},s_{3}\right\} & \left\{s_{4}\right\} & \left\{s_{3},s_{4},s_{5},s_{6}\right\} & \left\{s_{1},s_{2}\right\} \end{array}\right]$$

**Step 6.** Determine the comprehensive weights of experts. On the one hand, to reflect the preference of the expert, the different reputation and status of experts, the manager of the company can score the experts directly by allocating 100 scores to the five experts and then normalizing the score of each expert into 0 to 1 as the weights. In the scoring process, the minimum and maximum values for an expert are 10 and 30, respectively, to avoid oversized gaps among experts. Then, the subjective weight of experts is determined as $W^{S}=\left(0.16,0.18,0.22,0.26,0.18\right)$. On the other hand, to guarantee the consistency of the results, the objective weight of experts should be determined based on the consistency degree between each other. Based on the proposed method in Section 4.2, using Equations (2), (8) and (9), we can obtain the consistency degree of each alternative between any two experts. For example, the consistency degree matrix of alternative $p_{1}$ between any two experts is shown as follows.

$${\mathrm{CD}}_{1}=\left[\begin{array}{ccccc} 1 & 0.785 & 0.854 & 0.854 & 0.750\\ 0.785 & 1 & 0.917 & 0.889 & 0.938\\ 0.854 & 0.917 & 1 & 0.903 & 0.854\\ 0.854 & 0.889 & 0.903 & 1 & 0.896\\ 0.750 & 0.938 & 0.854 & 0.896 & 1 \end{array}\right]$$

Then, according to Equations (10) and (11), the averaging consistency degree and relative consistency degree among experts to each alternative are shown in Table 2.

Finally, according to Equations (12) and (13), we can obtain the objective weight of experts as $W^{O}=\left(0.201,0.201,0.199,0.202,0.197\right)$. The comprehensive weight of experts could be determined as $W=\left(0.180,0.190,0.210,0.231,0.189\right)$ by integrating the subjective weights with objective weights and setting α=0.5.

**Step 7.** Generate the PHFL group evaluation matrix. Based on the obtained comprehensive weight of experts and the operations for PHFLTS in Definition 9, the individual HFLTS-represented decision-making matrices could be integrated into one GDM matrix represented by PHFLTS. The result of integrated group decision-making matrix R is shown in Table 3.

**Step 8.** Derive the comprehensive weights of criteria. The weight of criteria should reflect both the subjective preference of experts and the objective information volume contained in the evaluation information for reasonable results. The subjective weight of criteria determined by BWM based on the evaluation of experts is $\psi^{S}=\left(0.382,0.128,0.073,0.417\right)$. The detailed process of using BWM to determine the subjective weight of criteria can be referred to in Appendix B. For determining the objective weight of criteria, the entropy-based method proposed in Section 4.2 is used here. In this study, we choose the third form of entropy measure for PHFLTS proposed in Definition 14 as the transition among distinct PHFLTS values are smoother than the others and, therefore, will enhance the interpretability of the calculation results. Then, according to Equation (14), we can obtain the entropy of each criterion under each alternative; the results are shown in Table 4. In addition, other objective weights determination methods such as the deviation/standard deviation methods can also be employed in accordance with specific application settings. The adoption of the entropy-based method presents its advantages of being convenient to use and making full use of the original information.

Based on the obtained entropy of each criterion, that is the last row of Table 4, the entropy-based objective weight of criteria according to Equation (15) is $\psi^{O}=\left(0.260,0.242,0.220,0.278\right)$. Finally, the comprehensive weight of experts could be determined as $\psi =\left(0.321,0.185,0.147,0.348\right)$ by integrating the subjective weights with objective weights and setting β=0.5. It can be seen that the criteria Equipment ($u_{4}$) and Human ($u_{1}$) are of high importance, contrary to the other two criteria. This result can provide effective references for HazMat transportation service providers, that is, to allocate more resources to improve the performance of Equipment ($u_{4}$) and Human ($u_{1}$) for decreasing the risks associated with HazMat transportation process. The results are consistent with our cognition and the research results of Zhao et al. [58] and Ambituuni et al. [63], which verifies the reliability of the results. Besides, it can be reasonably explained that the weight of Environment ($u_{3}$) is relatively low since the environment is usually objectively existing and cannot be largely affected by external factors. When concerned with the problem of transportation risk control, it is usually implied the purpose to reduce the risk probability and severity by taking into account several aspects of improvements. The objective criterion “Environment” cannot be changed manually, and, thus, it has relatively low importance rating. It can also be used to explain why Equipment ($u_{4}$) and Human ($u_{1}$) are of relatively high importance.

**Step 9.** Regenerate the PHFL group evaluation matrix. Based on the obtained comprehensive weight of experts and criteria, the group evaluation matrix should be re-generated by incorporating the weights into the initial evaluation information. The operations for PHFLTS defined in Definition 9 are used here. The re-generated group evaluation matrix is shown in Table 5.

**Step 10.** Rank alternatives using the proposed PHFL-TOPSIS method. For selecting the best HazMat transportation alternative, a ranking of all the alternatives should be determined. Considering the characteristics of HazMat transportation risk, all criteria in this study belong to cost type ($\Omega_{c}$). Therefore, the positive and negative solutions of each criterion should be the minimal and maximal one, respectively. The comparison method for PHFLTS proposed in Definition 11 is used here. Then, according to Equation (16), the positive and negative solutions are determined as follows.

$$P_{H_{S}}^{j+}=\left({\bar{P}}_{H_{S}}^{31},{\bar{P}}_{H_{S}}^{22},{\bar{P}}_{H_{S}}^{33},{\bar{P}}_{H_{S}}^{54}\right),P_{H_{S}}^{j-}=\left({\bar{P}}_{H_{S}}^{21},{\bar{P}}_{H_{S}}^{32},{\bar{P}}_{H_{S}}^{43},{\bar{P}}_{H_{S}}^{34}\right),$$

where the specific value of each element can be referred to in Table 5.

Then, according to Equation (17) and the distance measure for PHFLTS proposed in Definition 13, the positive distance and negative distance of each alternative can be calculated. The results are shown in Table 6. It is observed in Table 6 that the ranking of the alternatives is $p_{5}≻p_{2}≻p_{4}≻p_{3}≻p_{1}$ with Alternative $p_{5}$ considered as the best one. Therefore, the HazMat manufacturing company should choose the fifth transportation company who provided Alternative $p_{5}$ as the cooperator to transport the HazMat for the effort to minimize the transportation risk.

### 5.2. Weight Variation and Effect Analysis

In this subsection, we conduct the weight variation and effect analysis of the final ranking of the five HazMat transportation alternatives to the adjustments of experts and criteria weights.

**Figure 3:**
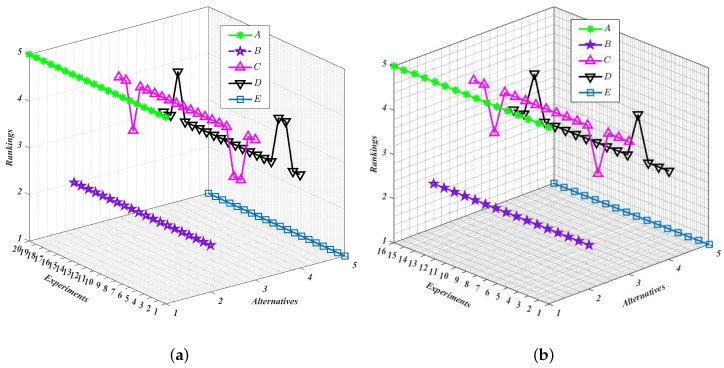
Weight variation and effect analysis: (**a**) weight variation and effect analysis on the final ranking of HazMat transportation alternatives to adjustments of the expert weights; and (**b**) weight variation and effect analysis on the final ranking of HazMat transportation alternatives to adjustments of the criteria weights.

On the one hand, we increase the weight of each expert by 20% and 40%, respectively, and then decrease them by 20% and 40%, respectively. Meanwhile, the weights of other experts are adjusted proportionally to guarantee that the sum of weights of all the experts still equals 1. In addition, we ensure that the initial inputs of the model and all its related parameters remain the same. Thus, for each expert, four experiments should be conducted. Finally, we can acquire 20 groups of results that are visualized in Figure 3a and listed in Table 7. With these data collected in this study, we can easily find that the ranking of HazMat transportation alternatives is not sensitive to the adjustment of expert weights, and the optimal alternative is always the fifth transportation company $p_{5}$.

On the other hand, we conduct similar experiments on each criterion. Then, there are 16 experiments to be conducted as only four criteria are involved for weight variation and effect analysis. The results of these experiments are visualized in Figure 3b and listed in Table 8. The slight changes of the lines in Figure 3b indicate that the adjustment of the criteria weights also pose a relatively small impact on the final ranking of HazMat transportation alternatives. The main reason behind this can be inferred to the original decision inputs, in which the evaluations on all criteria of all HazMat transportation alternatives show clear superiority over each other. For instance, Alternative $p_{5}$ is almost superior to the remaining alternatives in each criterion and each expert, which results that Alternative $p_{5}$ is always selected as the best one regardless of the change of the weights of experts and criteria. Moreover, the proposed PHFL-TOPSIS method focuses on the evaluations themselves rather than the outlier factors which might bring more subjectivity into the decision-making process.

Subsequently, to quantify the impact level of each adjustment on the weights of experts or attributes, we introduce Kendall’s tau distance [64] to measure the difference between the original ranking and each adjusted ranking. Let $\left[n\right]=\left\{1,\cdots ,n\right\}$ be a universe of elements. Let $S_{n}$ and $T_{n}$ be two different rankings on $\left[n\right]$ and for $\sigma_{S}\in S_{n}$ and $\sigma_{T}\in T_{n}$, let $\sigma_{S}\left(i\right)$ and $\sigma_{T}\left(i\right)$ denote the ranking of the element *i*, respectively. Then, the difference level $Diff\left(S_{n},T_{n}\right)$ between the two rankings $S_{n}$ and $T_{n}$ can be determined by $Diff\left(S_{n},T_{n}\right)=\sum_{\left(i,j\right):\sigma_{S}\left(i\right)>\sigma S\left(j\right)}1_{\left[\sigma_{T}\left(i\right)<\sigma_{T}\left(j\right)\right]}$, which can be used to quantify the difference level by measuring the total number of pairwise inversions.

Therefore, the impact level of each adjustment can be calculated by determining the difference levels between the original ranking and each adjusted rankings. The results obtained are presented in Table 7 and Table 8. In both Table 7 and Table 8, it can be observed that there exists no obvious connection between the expert weights and the difference level resulting from their adjustments. This also applies to the criteria weights adjustment scenarios, which implies that the proposed PHFL-TOPSIS method is robust to the exterior weight strategies adjustments, such as those defined in [65,66]. In this sense, the application of the novel group decision paradigm based on PHFTS in real-life scenario shows clearly its advantages. This fact is enhanced with the comparison analysis conducted in the next subsection.

### 5.3. Comparison Analysis

To further explore the superiority and effectiveness of the proposed PHFL-TOPSIS approach for HazMat transportation alternative evaluation and selection, a comparison is conducted on the same illustrative example in Section 5.1 with a method proposed by Farhadinia [67]. In this study, the evaluation information is expressed by HFLTS, the weights of criteria are completely unknown and then determined by entropy measure for HFLTS. Four steps are included in [67], which are set forth as below.

**Step 1.** Construct the decision-making matrix. An expert provides the evaluation of an alternative $p_{i}$ with respect to a criterion $u_{j}$ using HFLTS, and then the decision matrix is represented as $D={\left[H_{S}^{ij}\right]}_{m\times n}$.

**Step 2.** Determine the weights of criteria based on entropy measure for HFLTS. According to the decision matrix D; the entropy-based weights of criteria are determined as follows.

wj=1−Ejn−∑j=1nEj=1−∑i=1mEHSijEHSijnnn−∑j=1n∑i=1mEHSijEHSijnn,

where $E\left(H_{S}^{ij}\right)=1-\frac{2}{m}\sum_{i=1}^{m}\left(\frac{1}{L}\sum_{l=1}^{L}\frac{\delta_{il}}{g}\right)$ and $\delta_{il}$ represents the subscript of the l−th linguistic term corresponding to alternative $p_{i}$.

**Step 3.** Determine the positive solutions $H_{S}^{j+}$ and negative solutions $H_{S}^{j-}$ to establish $p^{+}$ and $p^{-}$.

$$H_{S}^{j+}=\left\{\begin{array}{c} \max_{1\leq i\leq m}H_{S}^{ij},j\in \Omega_{b}\\ \min_{1\leq i\leq m}H_{S}^{ij},j\in \Omega_{c} \end{array}\right.,P_{H_{S}}^{j-}=\left\{\begin{array}{c} \max_{1\leq i\leq m}PH_{S}^{ij},j\in \Omega_{c}\\ \min_{1\leq i\leq m}H_{S}^{ij},j\in \Omega_{b} \end{array}\right.,j=1,2,\cdots ,n$$

**Step 4.** Calculate the relative closeness coefficient $\eta \left(p_{i}\right)$ of each alternative $p_{i}$ to the positive solution.

$$\eta \left(p_{i}\right)=\frac{D\left(p_{i},p^{+}\right)}{D\left(p_{i},p^{+}\right)+D\left(p_{i},p^{-}\right)}=\frac{\sum_{j=1}^{n}w_{j}d\left(H_{S}^{ij},H_{S}^{j+}\right)}{\sum_{j=1}^{n}w_{j}d\left(H_{S}^{ij},H_{S}^{j+}\right)+\sum_{j=1}^{n}w_{j}d\left(H_{S}^{ij},H_{S}^{j-}\right)}$$

Then, the alternatives can be ranked according to $\eta \left(p_{i}\right)$.

**Remark** **1.** Farhadinia [67] pointed out that the higher is the $\eta \left(p_{i}\right)$, the better is the alternative. It is, from our view, incorrect because the numerator represents the distance between alternative $p_{i}$ to the positive solution $p^{+}$ when calculating $\eta \left(p_{i}\right)$. Therefore, we reach the opposite conclusion that the smaller is the $\eta \left(p_{i}\right)$, the better is the alternative. This conclusion can also be demonstrated by the idea of TOPSIS. The $\eta \left(p_{i}\right)$ in Ref. [67] is of the opposite meaning to $C_{i}^{*}$ in TOPSIS. Therefore, the appropriate conclusion is the smaller is the $\eta \left(p_{i}\right)$, the better is the alternative.

Applying the above four steps to the illustrative example in Section 5.1, the results can be obtained and are shown in Table 9.

**Figure 4:**
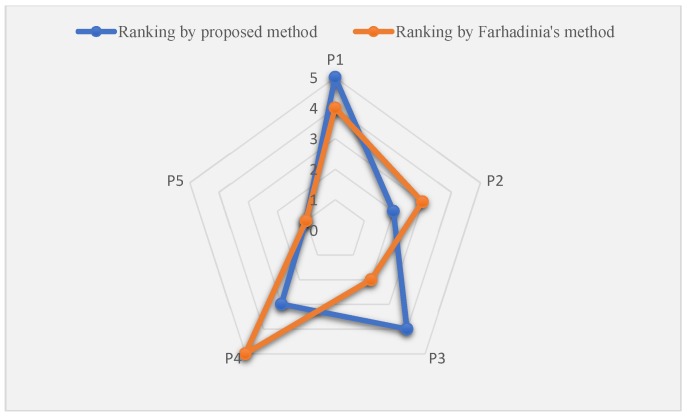
The ranking results of hazmats transportation alternatives with different approaches.

Based on the obtained results shown in Table 9, the ranking of alternatives with Fahardina’s method is $p_{5}≻p_{3}≻p_{2}≻p_{1}≻p_{4}$ and Alternative $p_{5}$ is the best one. According to the results in Table 6 and Table 9, the ranking results with different methods can be depicted in Figure 4.

It is worth noting that, compared with several other MCDM/MCGDM methods such as HFL-TOPSIS, HFL-VIKOR, and HFL-TODIM [68] dealing with the evaluation information expressed by HFLTS, the proposed method exhibits advantages in retaining the original information and deriving the reasonable weights of experts and criteria, and thus it guarantees the reliability of obtained results.

In Figure 4, it can be seen that both methods choose Alternative $p_{5}$ as the best alternative, which verifies the effectiveness of the proposed method. Nevertheless, the completed ranking of all alternatives is different with different methods. The reasons that lead to the difference are mainly located in three aspects. Firstly, Farhadinia’s method [67] is an MCDM method, in which only one single expert is involved in the decision-making process. However, due to the complexity and uncertainty of the decision-making environment, a decision-making team containing more than one expert should be established for collecting comprehensive evaluation information and making more reliable results. Moreover, the weights of experts are comprehensively determined in our study. Secondly, the weights of criteria are determined in different ways in the two methods. In Ref. [67], only the objective weights based on HFLTS-entropy are considered, while, in the extended PHFL-TOPSIS method proposed in this study, both the subjective weights and objective weights are considered, which in turn could yield more accurate results. Thirdly, in Ref. [67], HFLTS is used to express the linguistic evaluation of experts and derive the final ranking, while, in our proposed method PHFLTS is used, which considers the linguistic terms and proportional information simultaneously. The proportion could represent the support degree of the expert in GDM setting, and thus it is a more accurate information representation model. In summary, the proposed PHFL-TOPSIS method takes more factors that would influence the results into consideration. Compared with the existing MCDM method, our proposed MCGDM method contains more experts, and therefore could utilize more experience and knowledge of groups. Additionally, the PHFLTSs used in this study help to avoid information loss and therefore assure the accuracy of obtained results. Moreover, the comprehensive weights of criteria and experts are determined simultaneously, which can also increase the accuracy of the obtained results. Based on the analysis, the final ranking is derived by the proposed method with higher reliability.

## 6. Conclusions

Outsourcing the transportation business of a HazMat manufacturing enterprise is an effective way for manufacturing enterprises to decrease the risks and cost as well as to increase the core competitiveness for sustainable development by allocating the limited resources to the businesses that are of competitive superiority. In this regard, a critical issue worthy of increasing attention in the outsourcing decision is to evaluate the HazMat transportation alternatives and select the most desirable one. In this paper, we propose a novel integrated MCGDM method, namely, the PHFL-TOPSIS, to address the problem of evaluating and selecting the HazMat transportation alternatives. The main contributions and innovations of the proposed method are summarized below.

(1) This paper proposes several novel computational manipulations including the comparison laws, distance measure, similarity measure, and entropy measure for PHFLTS, which not only enrich the theory of PHFLTS but also enhance the applicability and effectiveness of PHFLTS.
(2) Two comprehensive weight assignment models are proposed in a bid to determine the comprehensive weights of experts and criteria in MCGDM contexts. Specifically, the objective weights of experts are determined on the basis of the similarity measure for PHFLTS; the objective weights of criteria are determined in the use of the entropy measure for PHFLTS. The obtained objective weights are then integrated with their subjective counterparts to derive comprehensive weights of experts and criteria. Taking the objective and subjective weights into consideration simultaneously could enhance the reasonability of decision-making effectively.
(3) The PHFL-TOPSIS method was developed on the basis of the defined distance measure for PHFLTS and the traditional TOPSIS method. The extended PHFL-TOPSIS method can deal with the situation in which the evaluation information is represented by PHFLTS, in which way it improved the applicability and accuracy of traditional TOPSIS method.
(4) A systematic framework has been proposed to address the problem of evaluating and selecting the HazMat transportation alternatives. During the decision-making process, the criteria used to evaluate the alternatives are firstly excavated, and their corresponding weights are then determined. The relative weight information provides an effective reference to control the risk during the HazMat transportation process. Eventually, a ranking of alternatives and the desirable alternative are determined. It provides the scientific decision and practical support for manager to decide the potential cooperator.

The feasibility and validity of the proposed method was verified by an illustrative example for choosing the most desirable HazMat transportation alternative of a HazMat manufacturing company. It is worth noting that the proposed computational manipulations can be automatically conducted by using the MATLAB R2019a, and thus it can be implemented conveniently in a wider range of applications. Moreover, the comparison analysis showed the advantages of the proposed method compared to a similar method, which implies that it succeeded in dealing with various decision settings more flexibly and comprehensively and derived more reliable results. The proposed systematic method can also be expanded to deal with other decision-making problems with similar characteristic. For example, it can be used into the fields of supplier selection, facility location evaluation and selection, water resource operation and management [69], municipal solid waste management [70], etc. Although the proposed PHFL-TOPSIS approach has exhibited certain superiorities, there are still several limitations. For example, when determining the weights of criteria, the interrelationship is overlooked. The ANP or Choquet integral can be used to deal with the problem. In addition, when comparing two PHFLTSs, the preference degree cannot be determined by the current study. To address this problem, probability-based comparison method for PHFLTS will be further developed in our continued research.

Our future attention will be devoted to the application of other soft computing/optimization techniques like Artificial Neural Network (ANN) [71], data envelopment analysis (DEA) [72], and computational intelligence-based methods [73] to study the problem of Hazmat transportation. Another direction is devoted to the development of novel aggregation strategies of PHFLTS referring to existing research on probabilistic hesitant fuzzy set [74], the PHFLTS-based preference relations elicitation, the consensus management of collective decision-making in HazMat transportation alternative evaluation [75,76,77], and the impact of data-driven HazMat transportation alternative evaluation and selection in the sustainable development of the environment and society.

## Figures and Tables

**Table 1:** Linguistic evaluation of alternatives with respect to each criterion.

		$u_{1}$	$u_{2}$	$u_{3}$	$u_{4}$
	$p_{1}$	Between M and H	At least M	M	Between M and H
	$p_{2}$	Greater than H	MH	Greater than M	At most M
$e_{1}$	$p_{3}$	Lower than M	VH	Between L and M	VH
	$p_{4}$	At least H	At least M	Between MH and VH	At most ML
	$p_{5}$	Between L and M	MH	At least M	Between L and ML
	$p_{1}$	Greater than MH	Between H and VH	Between L and MH	At least H
	$p_{2}$	At least MH	Lower than MH	M	Lower than M
$e_{2}$	$p_{3}$	Between L and ML	At least H	At most M	Between H and VH
	$p_{4}$	Between H and VH	M	At least H	Between L and M
	$p_{5}$	M	Between MH and H	M	VL
	$p_{1}$	At least MH	At least H	Between M and MH	Greater than M
	$p_{2}$	Between MH and VH	MH	MH	At most M
$e_{3}$	$p_{3}$	ML	VH	L	At least M
	$p_{4}$	Between MH and H	At least MH	At least H	Between M and MH
	$p_{5}$	Lower than ML	At least MH	Between H and VH	L
	$p_{1}$	H	Greater than H	M	H
	$p_{2}$	At least H	Between M and H	Between M and MH	L
$e_{4}$	$p_{3}$	Between L and M	H	Between L and M	Greater than MH
	$p_{4}$	Greater than MH	H	VH	Between L and M
	$p_{5}$	Between ML and M	MH	H	Lower than ML
	$p_{1}$	At least H	VH	At most M	At least H
	$p_{2}$	MH	M	At least H	Between VL and M
$e_{5}$	$p_{3}$	At least MH	Between H and VH	Between ML and M	H
	$p_{4}$	VH	H	Greater than MH	Lower than ML
	$p_{5}$	Between L and ML	Between MH and H	H	Between L and M

**Table 2:** The averaging and relative consistency degree among experts to each alternative.

	${AD}_{1}^{k}$	${AD}_{2}^{k}$	${AD}_{3}^{k}$	${AD}_{4}^{k}$	${AD}_{5}^{k}$	${RD}_{1}^{k}$	${RD}_{2}^{k}$	${RD}_{3}^{k}$	${RD}_{4}^{k}$	${RD}_{5}^{k}$
k=1	0.811	0.840	0.823	0.865	0.856	0.188	0.203	0.207	0.203	0.205
k=2	0.882	0.795	0.847	0.854	0.813	0.204	0.192	0.213	0.200	0.194
k=3	0.882	0.872	0.797	0.814	0.797	0.204	0.210	0.200	0.191	0.191
k=4	0.885	0.828	0.759	0.880	0.863	0.205	0.200	0.191	0.206	0.206
k=5	0.859	0.811	0.753	0.851	0.852	0.199	0.196	0.189	0.200	0.204

**Table 3:** PHFLTS-represented group decision-making matrix $R={\left[P_{H_{S}}^{ij}\right]}_{5\times 4}$.

	$u_{1}$
$p_{1}$	$\left\{\left(s_{3},0.06\right),\left(s_{4},0.130\right),\left(s_{5},0.551\right),\left(s_{6},0.260\right)\right\}$
$p_{2}$	$\left\{\left(s_{4},0.322\right),\left(s_{5},0.249\right),\left(s_{6},0.429\right)\right\}$
$p_{3}$	$\left\{\left(s_{0},0.06\right),\left(s_{1},0.232\right),\left(s_{2},0.442\right),\left(s_{3},0.077\right)\left(s_{4},0.063\right),\left(s_{5},0.063\right),\left(s_{6},0.063\right)\right\}$
$p_{4}$	$\left\{\left(s_{4},0.105\right),\left(s_{5},0.406\right),\left(s_{6},0.490\right)\right\}$
$p_{5}$	$\left\{\left(s_{0},0.105\right),\left(s_{1},0.165\right),\left(s_{2},0.270\right),\left(s_{3},0.460\right)\right\}$
	$u_{2}$
$p_{1}$	$\left\{\left(s_{3},0.045\right),\left(s_{4},0.045\right),\left(s_{5},0.245\right),\left(s_{6},0.665\right)\right\}$
$p_{2}$	$\left\{\left(s_{0},0.048\right),\left(s_{1},0.048\right),\left(s_{2},0.048\right),\left(s_{3},0.314\right),\left(s_{4},0.467\right),\left(s_{5},0.077\right)\right\}$
$p_{3}$	$\left\{\left(s_{5},0.421\right),\left(s_{6},0.580\right)\right\}$
$p_{4}$	$\left\{\left(s_{3},0.235\right),\left(s_{4},0.115\right),\left(s_{5},0.535\right),\left(s_{6},0.115\right)\right\}$
$p_{5}$	$\left\{\left(s_{4},0.671\right),\left(s_{5},0.260\right),\left(s_{6},0.070\right)\right\}$
	$u_{3}$
$p_{1}$	$\left\{\left(s_{3},0.060\right),\left(s_{4},0.130\right),\left(s_{5},0.551\right),\left(s_{6},0.260\right)\right\}$
$p_{2}$	$\left\{\left(s_{0},0.208\right),\left(s_{1},0.439\right),\left(s_{2},0.208\right),\left(s_{3},0.145\right)\right\}$
$p_{3}$	$\left\{\left(s_{3},0.053\right),\left(s_{4},0.053\right),\left(s_{5},0.452\right),\left(s_{6},0.443\right)\right\}$
$p_{4}$	$\left\{\left(s_{0},0.155\right),\left(s_{1},0.295\right),\left(s_{2},0.200\right),\left(s_{3},0.245\right),\left(s_{4},0.105\right)\right\}$
$p_{5}$	$\left\{\left(s_{0},0.306\right),\left(s_{1},0.479\right),\left(s_{2},0.153\right),\left(s_{3},0.063\right)\right\}$
	$u_{4}$
$p_{1}$	$\left\{\left(s_{0},0.047\right),\left(s_{1},0.095\right),\left(s_{2},0.095\right),\left(s_{3},0.611\right),\left(s_{4},0.153\right)\right\}$
$p_{2}$	$\left\{\left(s_{3},0.306\right),\left(s_{4},0.386\right),\left(s_{5},0.155\right),\left(s_{6},0.155\right)\right\}$
$p_{3}$	$\left\{\left(s_{0},0.048\right),\left(s_{1},0.318\right),\left(s_{2},0.202\right),\left(s_{3},0.202\right),\left(s_{5},0.231\right)\right\}$
$p_{4}$	$\left\{\left(s_{4},0.060\right),\left(s_{5},0.586\right),\left(s_{6},0.355\right)\right\}$
$p_{5}$	$\left\{\left(s_{3},0.235\right),\left(s_{4},0.276\right),\left(s_{5},0.339\right),\left(s_{6},0.150\right)\right\}$

**Table 4:** The entropy measure of each criterion under each alternative.

$E\left(P_{H_{S}}^{ij}\right)$	$u_{1}$	$u_{2}$	$u_{3}$	$u_{4}$
$p_{1}$	0.338	0.161	0.574	0.338
$p_{2}$	0.380	0.547	0.586	0.376
$p_{3}$	0.451	0.239	0.450	0.249
$p_{4}$	0.243	0.499	0.269	0.445
$p_{5}$	0.516	0.695	0.520	0.306
$E_{j}$	0.385	0.428	0.480	0.343

**Table 5:** Re-generated PHFLTS-represented group evaluation matrix ($\bar{R}={\left[{\bar{P}}_{H_{S}}^{ij}\right]}_{5\times 4}$).

	$u_{1}$
$p_{1}$	$\left\{\left(s_{1.197},0.06\right),\left(s_{1.783},0.130\right),\left(s_{2.624},0.551\right),\left(s_{6},0.260\right)\right\}$
$p_{2}$	$\left\{\left(s_{1.783},0.322\right),\left(s_{2.624},0.249\right),\left(s_{6},0.429\right)\right\}$
$p_{3}$	$\left\{\begin{array}{c} \left(s_{0},0.06\right),\left(s_{0.341},0.232\right),\left(s_{0.732},0.442\right),\left(s_{1.197},0.077\right)\\ \left(s_{1.783},0.063\right),\left(s_{2.624},0.063\right),\left(s_{6},0.063\right) \end{array}\right\}$
$p_{4}$	$\left\{\left(s_{1.783},0.105\right),\left(s_{2.624},0.406\right),\left(s_{6},0.490\right)\right\}$
$p_{5}$	$\left\{\left(s_{0},0.105\right),\left(s_{0.341},0.165\right),\left(s_{0.732},0.270\right),\left(s_{1.197},0.460\right)\right\}$
	$u_{2}$
$p_{1}$	$\left\{\left(s_{0.722},0.045\right),\left(s_{1.103},0.045\right),\left(s_{1.693},0.245\right),\left(s_{6},0.665\right)\right\}$
$p_{2}$	$\left\{\left(s_{0},0.048\right),\left(s_{0.199},0.048\right),\left(s_{0.434},0.048\right),\left(s_{0.722},0.314\right),\left(s_{1.103},0.467\right),\left(s_{1.693},0.077\right)\right\}$
$p_{3}$	$\left\{\left(s_{1.693},0.421\right),\left(s_{6},0.580\right)\right\}$
$p_{4}$	$\left\{\left(s_{0.722},0.235\right),\left(s_{1.103},0.115\right),\left(s_{1.693},0.535\right),\left(s_{6},0.115\right)\right\}$
$p_{5}$	$\left\{\left(s_{1.103},0.671\right),\left(s_{1.693},0.260\right),\left(s_{6},0.070\right)\right\}$
	$u_{3}$
$p_{1}$	$\left\{\left(s_{0},0.047\right),\left(s_{0.158},0.095\right),\left(s_{0.346},0.095\right),\left(s_{0.579},0.611\right),\left(s_{0.892},0.153\right)\right\}$
$p_{2}$	$\left\{\left(s_{0.579},0.306\right),\left(s_{0.892},0.386\right),\left(s_{1.385},0.155\right),\left(s_{6},0.155\right)\right\}$
$p_{3}$	$\left\{\left(s_{0},0.048\right),\left(s_{0.158},0.318\right),\left(s_{0.346},0.202\right),\left(s_{0.579},0.202\right),\left(s_{1.385},0.231\right)\right\}$
$p_{4}$	$\left\{\left(s_{0.892},0.060\right),\left(s_{1.385},0.586\right),\left(s_{6},0.355\right)\right\}$
$p_{5}$	$\left\{\left(s_{0.579},0.235\right),\left(s_{0.892},0.276\right),\left(s_{1.385},0.339\right),\left(s_{6},0.150\right)\right\}$
	$u_{4}$
$p_{1}$	$\left\{\left(s_{1.284},0.060\right),\left(s_{1.904},0.130\right),\left(s_{2.781},0.551\right),\left(s_{6},0.260\right)\right\}$
$p_{2}$	$\left\{\left(s_{0},0.208\right),\left(s_{0.368},0.439\right),\left(s_{0.789},0.208\right),\left(s_{1.284},0.145\right)\right\}$
$p_{3}$	$\left\{\left(s_{1.284},0.053\right),\left(s_{1.904},0.053\right),\left(s_{2.781},0.452\right),\left(s_{6},0.443\right)\right\}$
$p_{4}$	$\left\{\left(s_{0},0.155\right),\left(s_{0.368},0.295\right),\left(s_{0.789},0.200\right),\left(s_{1.284},0.245\right),\left(s_{1.904},0.105\right)\right\}$
$p_{5}$	$\left\{\left(s_{0},0.306\right),\left(s_{0.368},0.479\right),\left(s_{0.789},0.153\right),\left(s_{1.284},0.063\right)\right\}$

**Table 6:** The results of distance calculations.

	$u_{1}$	$u_{2}$	$u_{3}$	$u_{4}$		$u_{1}$	$u_{2}$	$u_{3}$	$u_{4}$	$D_{i}^{+}$	$D_{i}^{-}$	$C_{i}^{*}$	Rank
$d\left({\bar{P}}_{H_{S}}^{1j},P_{H_{S}}^{j+}\right)$	0.152	0.369	0.013	0.239	$d\left({\bar{P}}_{H_{S}}^{1j},P_{H_{S}}^{j-}\right)$	0.096	0.063	0.262	0.081	0.773	0.501	0.393	5
$d\left({\bar{P}}_{H_{S}}^{2j},P_{H_{S}}^{j+}\right)$	0.248	0.000	0.137	0.008	$d\left({\bar{P}}_{H_{S}}^{2j},P_{H_{S}}^{j-}\right)$	0.000	0.362	0.126	0.310	0.393	0.798	0.670	2
$d\left({\bar{P}}_{H_{S}}^{3j},P_{H_{S}}^{j+}\right)$	0.000	0.362	0.000	0.319	$d\left({\bar{P}}_{H_{S}}^{3j},P_{H_{S}}^{j-}\right)$	0.248	0.000	0.252	0.000	0.681	0.500	0.423	4
$d\left({\bar{P}}_{H_{S}}^{4j},P_{H_{S}}^{j+}\right)$	0.257	0.111	0.252	0.032	$d\left({\bar{P}}_{H_{S}}^{4j},P_{H_{S}}^{j-}\right)$	0.053	0.252	0.000	0.286	0.652	0.592	0.476	3
$d\left({\bar{P}}_{H_{S}}^{5j},P_{H_{S}}^{j+}\right)$	0.062	0.091	0.135	0.000	$d\left({\bar{P}}_{H_{S}}^{5j},P_{H_{S}}^{j-}\right)$	0.296	0.271	0.124	0.319	0.288	1.010	0.778	1

**Table 7:** Difference level between the original ranking and the adjusted ranking obtained based on the changing expert weights.

Experts/Weights		$e_{1}$(0.180)				$e_{2}$(0.190)				$e_{3}$(0.210)				$e_{4}$(0.231)				$e_{5}$(0.189)			
**The Alternatives**	**Original Ranking**	**+20%**	**+40%**	−**20%**	−**40%**	**+20%**	**+40%**	−**20%**	−**40%**	**+20%**	**+40%**	−**20%**	−**40%**	**+20%**	**+40%**	−**20%**	−**40%**	**+20%**	**+40%**	−**20%**	−**40%**
$p_{1}$	5	5	5	5	5	5	5	5	5	5	5	5	5	5	5	5	5	5	5	5	5
$p_{2}$	2	2	2	2	2	2	2	2	2	2	2	2	2	2	2	2	2	2	2	2	2
$p_{3}$	4	4	4	3	3	4	4	4	4	4	4	4	4	4	4	4	4	4	3	4	4
$p_{4}$	3	3	3	4	4	3	3	3	3	3	3	3	3	3	3	3	3	3	4	3	3
$p_{5}$	1	1	1	1	1	1	1	1	1	1	1	1	1	1	1	1	1	1	1	1	1
Difference level		0	0	1	1	0	0	0	0	0	0	0	0	0	0	0	0	0	0	0	0

**Table 8:** Difference level between the original ranking and each adjusted ranking obtained based on the changing criteria weights.

Criteria/Weights		$u_{1}$(0.382)				$u_{2}$(0.128)				$u_{3}$(0.073)				$u_{4}$(0.417)			
**The Alternatives**	**Original Ranking**	**+20%**	**+40%**	−**20%**	−**40%**	**+20%**	**+40%**	−**20%**	−**40%**	**+20%**	**+40%**	−**20%**	−**40%**	**+20%**	**+40%**	−**20%**	−**40%**
$p_{1}$	5	5	5	5	5	5	5	5	5	5	5	5	5	5	5	5	5
$p_{2}$	2	2	2	2	2	2	2	2	2	2	2	2	2	2	2	2	2
$p_{3}$	4	4	4	4	3	4	4	4	4	4	4	4	4	4	3	4	4
$p_{4}$	3	3	3	3	4	3	3	3	3	3	3	3	3	3	4	3	3
$p_{5}$	1	1	1	1	1	1	1	1	1	1	1	1	1	1	1	1	1
Difference level		0	0	0	1	0	0	0	0	0	0	0	0	0	1	0	0

**Table 9:** Calculation results of the illustrative example using the method of Farhadinia [67].

	$u_{1}$	$u_{2}$	$u_{3}$	$u_{4}$		$u_{1}$	$u_{2}$	$u_{3}$	$u_{4}$	$\eta \left(p_{i}\right)$	Rank
$d\left(H_{S}^{1j},H_{S}^{j+}\right)$	0.500	0.417	0.000	0.500	$d\left(H_{S}^{1j},H_{S}^{j-}\right)$	0.125	0.000	0.567	0.000	0.677	4
$d\left(H_{S}^{2j},H_{S}^{j+}\right)$	0.625	0.000	0.467	0.000	$d\left(H_{S}^{2j},H_{S}^{j-}\right)$	0.000	0.417	0.125	0.500	0.521	3
$d\left(H_{S}^{3j},H_{S}^{j+}\right)$	0.143	0.556	0.033	0.500	$d\left(H_{S}^{3j},H_{S}^{j-}\right)$	0.629	0.208	0.533	0.000	0.508	2
$d\left(H_{S}^{4j},H_{S}^{j+}\right)$	0.625	0.417	0.567	0.033	$d\left(H_{S}^{4j},H_{S}^{j-}\right)$	0.000	0.000	0.000	0.467	0.802	5
$d\left(H_{S}^{5j},H_{S}^{j+}\right)$	0.000	0.500	0.467	0.000	$d\left(H_{S}^{5j},H_{S}^{j-}\right)$	0.625	0.125	0.125	0.500	0.422	1

## Appendix A

**Table A1:** Completed HFLTS-represented decision-making matrix.

		$u_{1}$	$u_{2}$	$u_{3}$	$u_{4}$
	$p_{1}$	$\left\{s_{3},s_{4},s_{5}\right\}$	$\left\{s_{3},s_{4},s_{5},s_{6}\right\}$	$\left\{s_{3}\right\}$	$\left\{s_{3},s_{4},s_{5}\right\}$
	$p_{2}$	$\left\{s_{6}\right\}$	$\left\{s_{4}\right\}$	$\left\{s_{4},s_{5},s_{6}\right\}$	$\left\{s_{0},s_{1},s_{2},s_{3}\right\}$
$e_{1}$	$p_{3}$	$\left\{s_{0},s_{1},s_{2}\right\}$	$\left\{s_{6}\right\}$	$\left\{s_{1},s_{2},s_{3}\right\}$	$\left\{s_{6}\right\}$
	$p_{4}$	$\left\{s_{5},s_{6}\right\}$	$\left\{s_{3},s_{4},s_{5},s_{6}\right\}$	$\left\{s_{4},s_{5},s_{6}\right\}$	$\left\{s_{0},s_{1},s_{2}\right\}$
	$p_{5}$	$\left\{s_{1},s_{2},s_{3}\right\}$	$\left\{s_{4}\right\}$	$\left\{s_{3},s_{4},s_{5},s_{6}\right\}$	$\left\{s_{1},s_{2}\right\}$
	$p_{1}$	$\left\{s_{5},s_{6}\right\}$	$\left\{s_{5},s_{6}\right\}$	$\left\{s_{1},s_{2},s_{3},s_{4}\right\}$	$\left\{s_{5},s_{6}\right\}$
	$p_{2}$	$\left\{s_{4},s_{5},s_{6}\right\}$	$\left\{s_{0},s_{1},s_{2},s_{3}\right\}$	$\left\{s_{3}\right\}$	$\left\{s_{0},s_{1},s_{2}\right\}$
$e_{2}$	$p_{3}$	$\left\{s_{1},s_{2}\right\}$	$\left\{s_{5},s_{6}\right\}$	$\left\{s_{0},s_{1},s_{2},s_{3}\right\}$	$\left\{s_{5},s_{6}\right\}$
	$p_{4}$	$\left\{s_{5},s_{6}\right\}$	$\left\{s_{3}\right\}$	$\left\{s_{5},s_{6}\right\}$	$\left\{s_{1},s_{2},s_{3}\right\}$
	$p_{5}$	$\left\{s_{3}\right\}$	$\left\{s_{4},s_{5}\right\}$	$\left\{s_{3}\right\}$	$\left\{s_{0}\right\}$
	$p_{1}$	$\left\{s_{4},s_{5},s_{6}\right\}$	$\left\{s_{5},s_{6}\right\}$	$\left\{s_{3},s_{4}\right\}$	$\left\{s_{4},s_{5},s_{6}\right\}$
	$p_{2}$	$\left\{s_{4},s_{5},s_{6}\right\}$	$\left\{s_{4}\right\}$	$\left\{s_{4}\right\}$	$\left\{s_{0},s_{1},s_{2},s_{3}\right\}$
$e_{3}$	$p_{3}$	$\left\{s_{2}\right\}$	$\left\{s_{6}\right\}$	$\left\{s_{1}\right\}$	$\left\{s_{3},s_{4},s_{5},s_{6}\right\}$
	$p_{4}$	$\left\{s_{4},s_{5}\right\}$	$\left\{s_{4},s_{5},s_{6}\right\}$	$\left\{s_{5},s_{6}\right\}$	$\left\{s_{3},s_{4}\right\}$
	$p_{5}$	$\left\{s_{0},s_{1}\right\}$	$\left\{s_{4},s_{5},s_{6}\right\}$	$\left\{s_{5},s_{6}\right\}$	$\left\{s_{1}\right\}$
	$p_{1}$	$\left\{s_{5}\right\}$	$\left\{s_{6}\right\}$	$\left\{s_{3}\right\}$	$\left\{s_{5}\right\}$
	$p_{2}$	$\left\{s_{5},s_{6}\right\}$	$\left\{s_{3},s_{4},s_{5}\right\}$	$\left\{s_{3},s_{4}\right\}$	$\left\{s_{1}\right\}$
$e_{4}$	$p_{3}$	$\{s_{1},s_{2},s_{3}\}$	$\left\{s_{5}\right\}$	$\left\{s_{5}\right\}$	$\left\{s_{5},s_{6}\right\}$
	$p_{4}$	$\left\{s_{5},s_{6}\right\}$	$\left\{s_{5}\right\}$	$\left\{s_{5}\right\}$	$\left\{s_{1},s_{2},s_{3}\right\}$
	$p_{5}$	$\left\{s_{2},s_{3}\right\}$	$\left\{s_{4}\right\}$	$\left\{s_{4}\right\}$	$\left\{s_{0},s_{1}\right\}$
	$p_{1}$	$\left\{s_{5},s_{6}\right\}$	$\left\{s_{6}\right\}$	$\left\{s_{0},s_{1},s_{2},s_{3}\right\}$	$\left\{s_{5},s_{6}\right\}$
	$p_{2}$	$\left\{s_{4}\right\}$	$\left\{s_{3}\right\}$	$\left\{s_{5},s_{6}\right\}$	$\left\{s_{0},s_{1},s_{2},s_{3}\right\}$
$e_{5}$	$p_{3}$	$\left\{s_{4},s_{5},s_{6}\right\}$	$\left\{s_{5},s_{6}\right\}$	$\left\{s_{2},s_{3}\right\}$	$\left\{s_{5}\right\}$
	$p_{4}$	$\left\{s_{6}\right\}$	$\left\{s_{5}\right\}$	$\left\{s_{5},s_{6}\right\}$	$\left\{s_{0},s_{1}\right\}$
	$p_{5}$	$\left\{s_{2},s_{3}\right\}$	$\left\{s_{4},s_{5}\right\}$	$\left\{s_{5}\right\}$	$\left\{s_{1},s_{2},s_{3}\right\}$

## Appendix B

Detailed process of using BWM to determine the subjective weight of criteria.

**Step 1.** Determine a set of decision criteria. In this study, the decision criteria are the four identified risk criteria (i.e., Human ($u_{1}$), Management ($u_{2}$), Environment ($u_{3}$) and Equipment ($u_{4}$)) used to evaluate the transportation alternatives.

**Step 2.** Determine the most important and least important criteria. According to the preference of the decision-making team, Human ($u_{1}$) is regarded as the most important criterion while Environment ($u_{3}$) is regarded as the least important criterion.

**Step 3.** Determine the preference comparison of the best criterion over all the other criteria by pairwise comparison. We denote the Best to Others vector as

$$A_{B}=\left(a_{B1},a_{B2},\cdots ,a_{Bn}\right).$$

where $a_{Bj}$ represents the preference comparison of the best criterion $u_{B}$ over criterion $u_{j}$. Obviously, $a_{BB}=1$.

**Step 4.** Determine the preference comparison of all the criteria over the worst criterion. Similar to step 3, we denote the Others to Worst vector as

$$A_{W}={\left(a_{1W},a_{2W},\cdots ,a_{nW}\right)}^{T}.$$

where $a_{jW}$ represents the preference comparison of the criterion $u_{j}$ over the best criterion $u_{B}$. Obviously, $a_{WW}=1$.

Specifically, in this study, the decision-making team provides the $A_{B}=\left(1,3,6,1\right)$ and $A_{W}\;=\;\left(5,2,1,6\right)$.

**Step 5.** Define the optimal weights $\left(w_{1},w_{2},\cdots ,w_{n}\right)$. For derive the optimal weights, the following model is established

$$\begin{array}{c} \min\max\left\{\left|\frac{w_{B}}{w_{j}}-a_{Bj}\right|,\left|\frac{w_{j}}{w_{W}}-a_{jW}\right|\right\}\\ s.t.\left\{\begin{array}{c} \sum_{j=1}^{n}w_{j}=1\\ w_{j}\geq 0,j=1,2,\cdots ,n \end{array}\right. \end{array}$$

Then, to solve the above model, it can be equally transferred to the following model:

$$\begin{array}{c} \min\xi \\ s.t.\left\{\begin{array}{c} \left|\frac{w_{B}}{w_{j}}-a_{Bj}\right|\leq \xi ,j=1,2,\cdots ,n\\ \left|\frac{w_{j}}{w_{W}}-a_{jW}\right|\leq \xi ,j=1,2,\cdots ,n\\ \sum_{j=1}^{n}w_{j}=1\\ w_{j}\geq 0,j=1,2,\cdots ,n \end{array}\right. \end{array}$$

Solving the above model, the optimal solution of weights $\left(w_{1},w_{2},\cdots ,w_{n}\right)$ and $\xi^{*}$ can be obtained.

In this study, based on the preference comparison vectors $A_{B}$ and $A_{W}$, the following model used to derive the optimal weights of criteria could be established.

$$\begin{array}{c} \min\xi \\ s.t.\left\{\begin{array}{c} -\xi *w_{1}\leq w_{4}-w_{1}\leq \xi *w_{1}\\ \begin{array}{c} -\xi *w_{2}\leq w_{4}-3w_{2}\leq \xi *w_{2}\\ -\xi *w_{3}\leq w_{4}-6w_{3}\leq \xi *w_{3}\\ -\xi *w_{3}\leq w_{1}-5w_{3}\leq \xi *w_{3}\\ -\xi *w_{3}\leq w_{2}-2w_{3}\leq \xi *w_{3} \end{array}\\ \sum_{j=1}^{4}w_{j}=1\\ w_{j}\geq 0,j=1,2,\cdots ,4 \end{array}\right. \end{array}$$

Solving the model, we have $W^{S}=\left(0.382,0.128,0.073,0.417\right)$ and $\xi^{*}=0.256$. Then, the consistency ratio is 0.085 (i.e., 0.2560.25633=0.085), which is smaller than 0.1 and thus pass the consistency test. The subjective weights of criteria are determined.
